# Cuproptosis facilitates immune activation but promotes immune escape, and a machine learning–based cuproptosis‐related signature is identified for predicting prognosis and immunotherapy response of gliomas

**DOI:** 10.1111/cns.14380

**Published:** 2023-07-28

**Authors:** Shi Feng, Yonggang Zhang, Hua Zhu, Zhihong Jian, Zhi Zeng, Yingze Ye, Yina Li, Daniel Smerin, Xu Zhang, Ning Zou, Lijuan Gu, Xiaoxing Xiong

**Affiliations:** ^1^ Department of Neurosurgery Renmin Hospital of Wuhan University Wuhan China; ^2^ Department of Pathology Renmin Hospital of Wuhan University Wuhan China; ^3^ Department of Anesthesiology Renmin Hospital of Wuhan University Wuhan China; ^4^ Department of Neurosurgery University of Texas Health Science Center at San Antonio San Antonio Texas USA; ^5^ Department of Radiation Oncology, Hubei Cancer Hospital, Tongji Medical College Huazhong University of Science and Technology Wuhan China; ^6^ Central Laboratory Renmin Hospital of Wuhan University Wuhan China

**Keywords:** cuproptosis, gliomas, machine learning, tumor immune microenvironment

## Abstract

**Aims:**

Cell death, except for cuproptosis, in gliomas has been extensively studied, providing novel targets for immunotherapy by reshaping the tumor immune microenvironment through multiple mechanisms. This study aimed to explore the effect of cuproptosis on the immune microenvironment and its predictive power in prognosis and immunotherapy response.

**Methods:**

Eight glioma cohorts were included in this study. We employed the unsupervised clustering algorithm to identify novel cuproptosis clusters and described their immune microenvironmental characteristics, mutation landscape, and altered signaling pathways. We verified the correlation among FDX1, SLC31A1, and macrophage infiltration in 56 glioma tissues. Next, based on multicenter cohorts and 10 machine learning algorithms, we constructed an artificial intelligence–driven cuproptosis‐related signature named CuproScore.

**Results:**

Our findings suggested that glioma patients with high levels of cuproptosis had a worse prognosis owing to immunosuppression caused by unique immune escape mechanisms. Meanwhile, we experimentally validated the positive association between cuproptosis and macrophages and its tumor‐promoting mechanism in vitro. Furthermore, our CuproScore exhibited powerful and robust prognostic predictive ability. It was also capable of predicting response to immunotherapy and chemotherapy drug sensitivity.

**Conclusions:**

Cuproptosis facilitates immune activation but promotes immune escape. The CuproScore could predict prognosis and immunotherapy response in gliomas.

## Graphical abstract

We employed the unsupervised clustering algorithm to identify novel cuproptosis clusters and described their immune microenvironmental characteristics, mutation landscape, and altered signaling pathways. A machine learning–based cuproptosis‐related signature, named CuproScore, was constructed based on the eight independent gliomas cohorts and ten machine‐learning algorithms. It provides a well‐developed, stable, and robust model for predicting the prognosis and immunotherapy response in glioma.

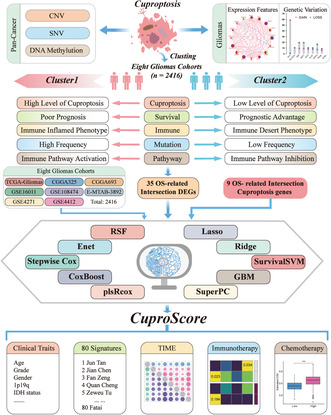

## 1 INTRODUCTION

Gliomas are among the most prevalent malignant tumors of the central nervous system.^1^ In 2021, the World Health Organization (WHO) assigned four pathological grades to gliomas. Notably, patients with low‐grade gliomas (LGG), WHO grades II and III, have an overall survival (OS) of 8–10 years. Additionally, WHO grade IV is assigned to glioblastoma. Owing to its aggressiveness and resistance to chemotherapeutic agents,^2^ the OS of patients with glioblastoma treated with postoperative adjuvant radiotherapy is only 12–14 months.^3^ The comprehensive treatment options for gliomas include surgery, radiotherapy, and chemotherapy.^4^ However, the prognosis of glioma remains poor, necessitating the search for novel therapeutic targets. Furthermore, significant epigenetic, genetic, and microenvironmental variations in glioma make it highly resilient and explain its multiple mechanisms of therapeutic resistance.^5^ Notably, alterations in the tumor immune microenvironment (TIME) can reportedly influence glioma proliferation, migration, and progression.^6^ Immunotherapy, represented by immune checkpoint inhibitors (ICI), is emerging as an effective treatment strategy for solid tumors.^7^ However, gliomas have a unique immune microenvironment characterized by a high frequency of myeloid cells, high macrophage predominance, low frequency of T cells, and low levels of cell surface inhibitory markers. These characteristics enable immune escape reducing ICI effectiveness in gliomas.^8^, ^9^ Although some candidate biomarkers, such as immune checkpoint expression,^10^ tumor mutational burden (TMB),^11^ and microsatellite instability (MSI),^12^ can predict ICI efficacy in tumor patients, their application has limitations. Therefore, identifying new targets for glioma treatment and establishing an individualized prognostic assessment system in combination with TIME can aid in predicting patient prognosis, investigating drug resistance mechanisms, and developing individualized treatment strategies.

Cuproptosis is an innovative cell death mechanism distinct from apoptosis, ferroptosis, necroptosis, and pyroptosis.^13^ It occurs when the lipid‐acylated components of the tricarboxylic acid cycle in the mitochondria bind directly to overloaded copper, leading to the loss of iron–sulfur proteins and abnormal accumulation of lipid‐acylated proteins, resulting in proteotoxic stress and cell death. Notably, the microenvironment of the human body depends heavily on copper for homeostasis, and copper‐ion balance in the mitochondria is implicated in many diseases, including neurodegenerative, metabolic, genetic, and cardiovascular diseases.^14^ Furthermore, copper can act as a signal to activate the immune system, forming a defense response.^15^ Cancer patients have higher blood and tissue copper levels than healthy individuals,^16^, ^17^ inhibiting copper transport hinders cancer cell proliferation.^18^ Moreover, copper affects PD‐L1 expression in tumors and influences tumor immune escape,^19^ and glioblastoma cells use the tricarboxylic acid cycle/oxidative phosphorylation differently from normal brain tissues.^20^ Overall, mitochondrial dysfunction is essential in glioma development, progression, and drug resistance.^21^, ^22^, ^23^

Although copper and mitochondrial homeostases have complex implications for tumorigenesis and progression, the role of cuproptosis in tumors remains unknown. Only a few studies have investigated the relationship between cuproptosis‐related molecules and gliomas. Zhang et al.^24^ constructed a cuproptosis risk model based on 10 cuproptosis genes. Moreover, Wang et al.^25^ identified two novel glioma cuproptosis phenotypes and developed a differential gene‐based risk model. However, these studies did not demonstrate the multiomic characteristics associated with cuproptosis genes and lacked large multicenter cohorts to strictly validate their signatures. Additionally, the selection of algorithms by researchers may rely largely on their preferences and biases. Consequently, it is imperative to comprehensively analyze cuproptosis patterns in multicenter cohorts from a multiomics perspective and explore the role of cuproptosis regulatory molecules in gliomas regarding TIME, epigenetic mutations, and immunotherapy.

In this study, we collected 16 cuproptosis‐associated genes and demonstrated their transcriptomic and epigenetic mutational heterogeneity in pan‐cancer. Using unsupervised clustering, we identified two cuproptosis expression patterns correlated to cuproptosis with TIME, somatic mutations, and copy number variation in gliomas. Moreover, we explored the tumor‐promoting mechanism of *FDX1* in U251 and A172 cells. Subsequently, 10 machine‐learning algorithms were enrolled, transformed into 117 combinations, and performed on 8 independent glioma cohorts. Next, we constructed and validated a multicenter machine learning–based cuproptosis‐related signature, CuproScore, with the best performance among the 117 models. The results of the 8 cohorts consistently indicated that the CuproScore had a stable and robust predictive performance for survival outcomes, superior to common clinical traits and 80 published signatures. Moreover, it could predict the responses to immunotherapy and chemotherapy drug sensitivity. Overall, CuproScore could be a promising tool for developing new treatment strategies for more individualized and precise medicine.

## 2 MATERIALS AND METHODS

### 2.1 Acquisition and preprocessing of data

The workflow of the study is shown in Figure S1. Details of data acquisition and preprocessing can be found in the Appendix S1.

### 2.2 Unsupervised clustering of cuproptosis genes

Sixteen cuproptosis genes were obtained from previous research.^13^, ^26^, ^27^ The eight cohorts and meta‐cohort were clustered unsupervised using the unsupervised average linkage K‐means clustering analysis^28^, ^29^ and repeated 1000 times to ensure classification stability.^30^ We then used principal component analysis (PCA) to validate the clustering based on the expression profiles of the above genes.

### 2.3 Development of the cuproscore prognostic model

We constructed a machine learning–based cuproptosis‐related signature, CuproScore, with the best performance among the 117 combinations. The detailed methods of this part are provided in the Appendix S1.

### 2.4 Statistical analysis

All statistical analyses were performed by R software (version 4.0.2). Procedure details are provided in the Appendix S1.

Other bioinformatics methods and experimental methods are provided in the Appendix S1.

## 3 RESULTS

### 3.1 Transcriptional and genetic characteristics of 16 cuproptosis genes

We summarized the data on 16 cuproptosis genes from previous studies,^13^, ^26^, ^27^ including *FDX1*, *LIAS*, *LIPT1*, *DLD*, *DLAT*, *PDHA1*, *PDHB*, *MTF1*, *GLS*, *CDKN2A*, *SLC31A1*, *ATP7B*, *ATP7A*, *DBT*, *GCSH*, and *DLST*. Next, we analyzed the cuproptosis‐related single nucleotide variant (SNV) data to investigate the variant types and frequencies of the cuproptosis molecules in every cancer species. As shown in the percentage heatmap, UCEC, SKCM, NHSC, and PAAD had the highest deleterious mutation frequencies among all cancer types; however, SNV frequencies in glioblastoma and LGG were <6% (Figure S2A). Furthermore, the top 10 cuproptosis molecules with the highest SNV frequency in pan‐cancer tissues were identified using a waterfall diagram. Among the 1024 samples, 966 had mutated cuproptosis molecules at a frequency of 94.34%. Additionally, *CDKN2A* had the highest mutation frequency (38%), followed by *ATP7A* (19%) and *ATP7B* (17%), with the missense mutations being the main SNV type (Figure S2B). We confirmed that genetic diversity significantly affected cuproptosis molecule expression. Moreover, copy number variation (CNV) and mRNA expression levels of cuproptosis molecules were positively correlated in most cancer types, including LGG and glioblastoma (Figure S2C). CNV frequency analysis revealed that the CNV of 16 cuproptosis molecules differed significantly in pan‐cancer tissues, with *DLD* and *DLAT* having the highest frequency of CNV in LGG and glioblastoma (Figure S3A), consisting primarily of copy number heterozygous amplification and deletion. Furthermore, homozygous CNV was less common, and only *CDKN2A* had homozygous deletions (Figure S3B, C). Comparing the correlation between DNA methylation levels and mRNA expression showed that the correlation in most cancer specimens was significantly negative. However, this association was weak in the LGG and glioblastoma (Figure S2D). Cuproptosis gene expression was highly variable in tumor samples from various cancer types, indicating that the mRNA expression imbalance of cuproptosis molecules was significantly associated with pan‐cancer genomic variance.

In this study, we focused on the effects of cuproptosis on gliomas. In the Cancer Genome Atlas (TCGA) database, among 610 samples, only 19 had mutated cuproptosis molecules at a frequency of 3.11%, suggesting that SNV of cuproptosis genes might not be the dominant cause of expression imbalance. Among these 19 patients, *ATP7A* and *CDKN2A* had the highest mutation frequencies; however, only 1% had missense mutations (Figure S2E). Moreover, co‐mutations between *ATP7A* and *CDKN2A*, *ATP7B* and *GLS*, and *CDKN2A* and *GLS* were significant (*p* < 0.05; Figure S2G). Next, CNV was common among the 16 cuproptosis genes. The CNV of *DLD* increased, and that of *ATP7B*, *DLST*, *CDKN2A*, and *SLC31A*1 decreased significantly (Figure S2F). The locations of cuproptosis genes with CNVs on the chromosomes are shown in the Circle Map (Figure S2H). According to these results, CNV, rather than SNV, is the primary cause of the imbalance of cuproptosis molecules in gliomas.

Most of the regulators showed significant differences in expression between normal and malignant glioma tissues. Only *GLS* and *ATP7B* had low expression levels in glioma patients; however, others, including *FDX1*, *LIPT1*, *DLD*, and *SLC31A1*, were overexpressed (Figure S4B). Additionally, the expression levels of *FDX1*, *LIPT1*, *DLD*, *DLAT*, *SLC31A1*, *ATP7B*, *ATP7A*, *GCSH*, and *DLST* varied considerably in different WHO grades from II to IV (Figure S4C). The panoramic view of the prognostic value and interactions among cuproptosis molecules is depicted in the network (Figure S4A); most cuproptosis genes were positively correlated. Furthermore, Cox regression analysis in the TCGA database showed that *FDX1*, *LIPT1*, *DLD*, *DLAT*, *SLC31A1*, *ATP7A*, and *DLST* might be risk factors; however, *LIAS*, *ATP7B*, and *GCSH* were significant preventive factors for gliomas (Figure S2I). Owing to the significant differences in transcription profiles and genomic variants, cuproptosis imbalance contributes to tumorigenesis and progression in patients with glioma.

### 3.2 Identification of novel cuproptosis patterns by unsupervised clustering

**FIGURE 1:**
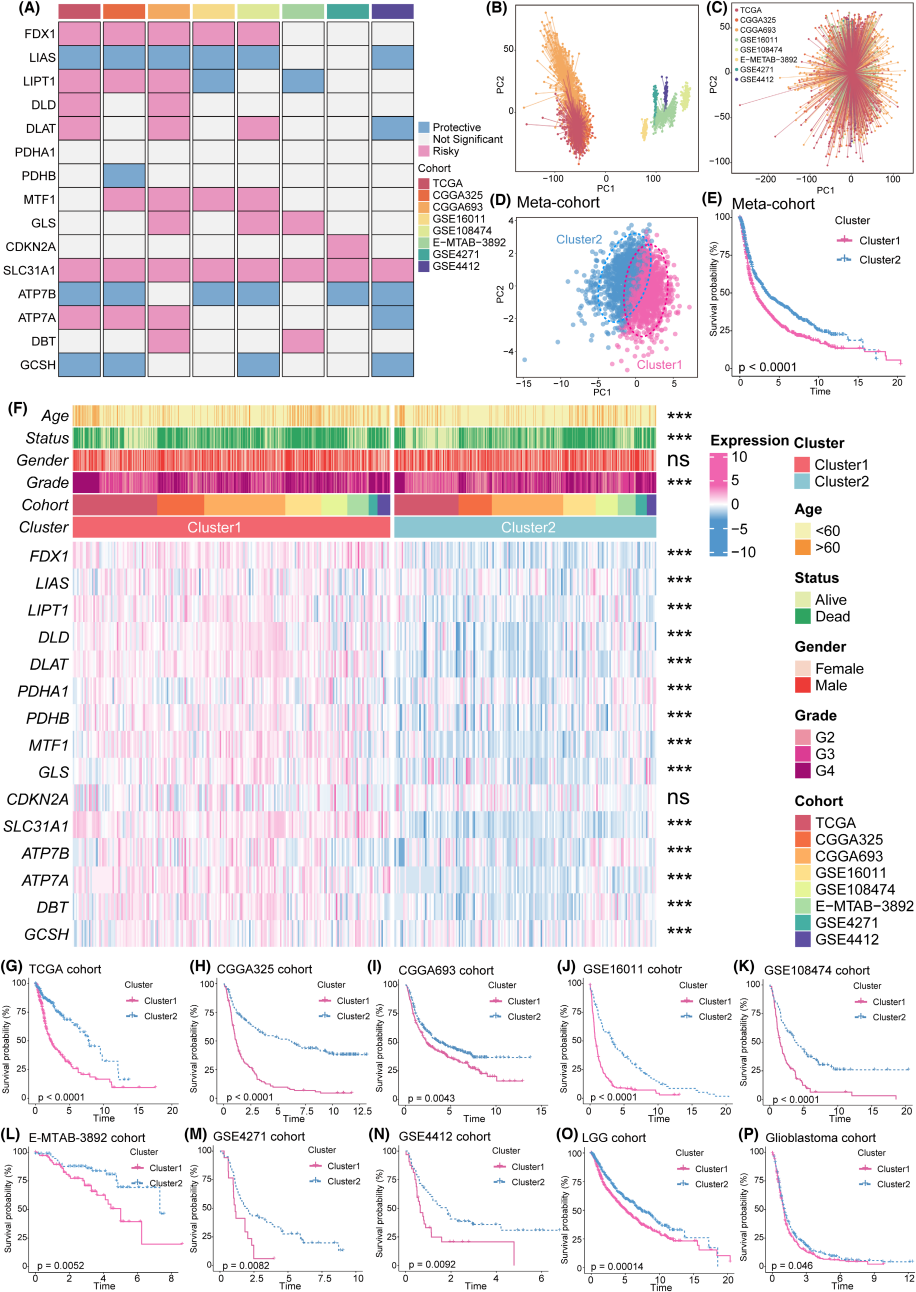
Identification of two cuproptosis subgroups by unsupervised clustering based on k‐means. (A) This heatmap demonstrates the prognostic value of cuproptosis‐related genes in eight cohorts. (B, C) PCA shows expression baseline before (B) and after (C) removing the batch effect of eight cohorts. (D) PCA for the transcriptome profiles of cuproptosis patterns in the meta‐cohort. (E) The log‐rank test and Kaplan–Meier curves revealed significant survival rate differences between the two cuproptosis clusters in the meta‐cohort. (F) This heatmap demonstrates the relationships between the two cuproptosis phenotypes, clinicopathologic characteristics, and the expression variations in the cuproptosis‐related genes. The top portion represented Fisher's precise test. The lower portion indicated the Wilcoxon rank‐sum test. ***p < 0.001, **p < 0.01, *p < 0.05, and “ns” stands for no statistical significance. (G‐P) The log‐rank test and Kaplan–Meier curves revealed significant survival rate differences between the two cuproptosis clusters in the TCGA (G), CGGA325 (H), CGGA693 (I), GSE16011 (J), GSE108474 (K), E‐MTAB‐3892 (L), GSE4271 (M), GSE4412 (N), LGG cohort (O), and glioblastoma cohort (P).

To comprehensively understand the modulation of gliomas by cuproptosis, we collected 2416 patients in 8 independent glioma cohorts. Univariate Cox analysis was performed on 16 cuproptosis genes in all 8 cohorts (Figure 1A). We considered cuproptosis genes possessing *p* < 0.05 for more than four cohorts as stable prognostically significant cuproptosis molecules, including *FDX1*, *LIAS*, *LIPT1*, *DLAT*, *MTF1*, *SLC31A1*, *ATP7B*, *ATP7A*, and *GCSH*. Then, we combined the eight cohorts into an integrated meta‐cohort and removed the batch effect using the “Combat” algorithm. The PCA algorithm visualized the expression baseline before (Figure 1B) and after (Figure 1C) removing the batch effect, indicating the batch effect had been effectively corrected. To identify potential cuproptosis phenotypes of gliomas. We applied K‐means–based unsupervised clustering in eight independent cohorts and the meta‐cohort based on the nine prognostically significant cuproptosis genes. Unsupervised clustering with the R package “ConsensusClusterPlus” identified two unique cuproptosis clusters, termed Cluster1 and Cluster2. The PCA algorithm confirmed that patients in the meta‐cohort could be distinguished perfectly by cuproptosis molecules **(**Figure 1D). Similar results were obtained in all eight independent cohorts (Figure S5A–H). Given that low‐grade and higher‐grade gliomas may have different expression patterns, we extract the LGG and glioblastoma cohorts from the meta‐cohort. Then, we conducted unsupervised clustering in the LGG and glioblastoma cohorts, respectively, and obtained the same results (Figure S5I,J). The molecular discrepancies in cuproptosis between both clusters are shown in heatmaps (Figure 1F; Figure S5K). The cuproptosis molecules in Cluster 1 showed significantly higher expression levels in almost all eight cohorts. The biased distribution of cuproptosis level was also obtained in both LGG and glioblastoma cohorts (Figure S5K). Additionally, Clusters 2 had more patients with lower grades of glioma (grades II or III). Patients in Cluster 1 were older than Cluster 2 patients (Figure 1F; Figure S5K). Survival analysis of both cuproptosis patterns demonstrated a relatively significant prognostic advantage in Cluster 2 in all eight cohorts (Figure 1G–N), which was also obtained in the LGG and glioblastoma cohorts (Figure 1O,P). Based on the univariate and multivariate Cox analyses, Cluster 2 was a significant independent prognostic factor in almost all cohorts except TCGA, GSE4271, and GSE4412 (Table S1).

### 3.3 Characterization of TIME immune variations in different cuproptosis patterns

Since tumor progression and killing are inextricably connected to immunity, we investigated the differences in TIME between distinct cuproptosis clusters. We focused on the TCGA cohort, which had comprehensive clinical information and multiple omics data. Meanwhile, to ensure the comprehensiveness of the analysis, we also conducted parallel analyses in the meta‐cohort, LGG cohort, and glioblastoma cohort.

**FIGURE 2:**
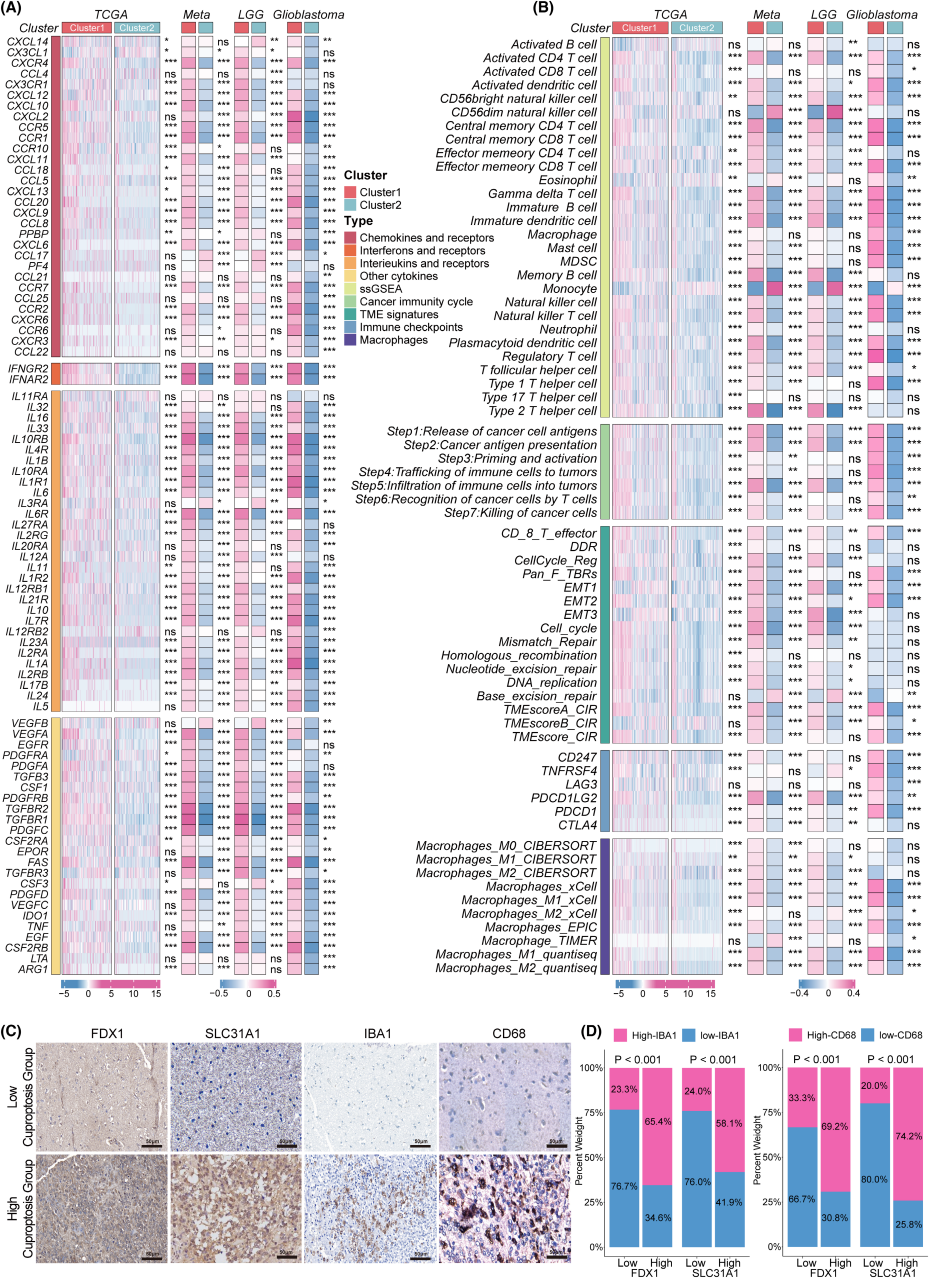
Alterations of immunomodulators and immune infiltration between two cuproptosis subgroups. (A) This heatmap displays alterations in the mRNA expression levels of chemokines, interleukins, interferons, and their corresponding receptors, and other cytokines between the two cuproptosis subtypes in the TCGA cohort, meta‐cohort, LGG cohort, and glioblastoma cohort. The asterisks denoted the p‐values (***p < 0.001, ** *p* < 0.01, * *p* < 0.05) for the Wilcoxon rank‐sum test. (B) This heatmap displays alterations in the immune infiltrating cell types, the enrichment levels of seven anti‐tumor immune cycle steps, TIME signatures, immune checkpoints, and macrophages calculated by other algorithms between the two cuproptosis subtypes in the TCGA cohort, meta‐cohort, LGG cohort, and glioblastoma cohort. The asterisks denoted the p‐values (***p < 0.001, ** *p* < 0.01, * *p* < 0.05) for the Wilcoxon rank‐sum test. (C) Representative IHC staining images of FDX1, SLC31A1, IBA1, and CD68 in human glioma tissues (*n* = 56, scale bars: 50 μm). (D) Semi‐quantitative analyses of IHC staining of IBA1 and CD68 expression in low‐ and high‐cuproptosis groups of 56 gliomas tissues.

Initially, we explored the differences in immune checkpoints and immunomodulators between cuproptosis clusters. The effects of cuproptosis on TIME revealed a higher expression of chemokines in Cluster 1 in TCGA, meta, LGG, and glioblastoma cohorts, such as *CXCL12*, *CXCL10*, *CCR5*, *CCR10*, *CCL5*, and *CCR2* (Figure 2A), attracting immunosuppressive cells, such as Tregs, macrophages, myeloid‐derived suppressor cells (MDSCs), and monocytes, vital in immune escape. Cluster 1 showed increased expression of interferons, interleukins, receptors, and other cytokines. However, Cluster 2 showed significantly lower levels of immunomodulators (Figure 2A).

Next, we used the single‐sample GSEA (ssGSEA) algorithms to calculate the overall infiltration abundance in TCGA, meta, LGG, and glioblastoma cohorts. Investigation of gene signatures indicated a greater number of immune cells with significant immunosuppressive function in Cluster 1 in all cohorts, such as type 2 T helper cells, MDSCs, regulatory T cells, and macrophages. Interestingly, activated CD8+ T cells, activated B cells, activated dendritic cells, mast cells, and NK cells were also abundant in Cluster 1, suggesting that the immune cells dominating both immune escape and anti‐tumor functions in Cluster 1 were in an activated state (Figure 2B). To ensure the stability of our results, we calculated the immune infiltration abundance of macrophages, which are among the most abundant immune cells in gliomas, using five other algorithms. Cluster 1 showed significantly higher levels of macrophages in almost all algorithms (Figure 2B). Furthermore, anti‐tumor immunity must complete a cycle of steps termed the cancer immune cycle to kill cancer cells effectively. The scores for the seven immune cycle steps were calculated using ssGSEA. All steps scored higher in Cluster 1 but showed deficient levels in Cluster 2, verifying the crucial effects of cuproptosis in TIME (Figure 2B). Biomarkers of several broadly acknowledged immune pathways were also investigated. Cluster 1 had significantly higher immune‐related gene set scores than Cluster 2 in TCGA, meta, and LGG cohorts. Cluster 1 exhibited a higher expression of six immune checkpoints than Cluster 2, including *PDCD1*, *CD247*, *TNFRSF4*, *LAG3*, *PDCD1LG2*, and *CTLA4* (Figure 2B). This result suggests that Cluster 1 expressed higher levels of immune checkpoint molecules than Cluster 2 to avoid immune killing after immune activation.

Subsequently, the TIDE algorithm and ESTIMATE method were adopted to compare the differences in TIME between the two cuproptosis patterns. Cluster 1 had higher stromal and immune scores and lower tumor purity than Cluster 2, suggesting a relatively hot TIME (Figure S6D–F). Next, we discovered Cluster 1 that showed a higher TIDE score, exclusion scores, MDSCs, CAF, IFN, SNV, neoantigens, lower dysfunction score, MSI, and CTA score (Figure S6G–O), consistent with the above results and possibly implicated in immune escape in Cluster 1. Moreover, in the analysis of TIME signatures, Cluster 1 had higher levels of innate immunity, priming activation, T cells, IFN‐γ response, Treg, MDSC, recognition of tumor, proliferation, and glycolysis (Figure S6B,C). Using a correlation method, several cuproptosis regulatory genes, including FDX1, SLC31A1, ATP7A, LIPT1, CDKN2A, and DLST, were positively associated with most immunosuppressive cells (Figure S6A). Immunohistochemistry (IHC) staining was further conducted with 56 glioma tissues. FDX1 and SLC7A11 (hub regulators of cuproptosis) staining was performed to evaluate cuproptosis status. We found that IBA1 and CD68 (markers of macrophages) were significantly upregulated in high cuproptosis group, which confirmed that patients with gliomas in a high level of cuproptosis were characterized by macrophage enrichment (Figure 2C,D). Although patients with high expression of cuproptosis molecules have higher immune infiltration than those without it, the presence of immunoinhibitory cells, high concentrations of immunoinhibitory cytokines, high expression of immune checkpoints, a high TIDE score, and a high level of CAF may cause immune escape in Cluster 1, consistent with the poor prognosis of Cluster 1 patients.

### 3.4 Characteristics of genome alterations between cuproptosis phenotypes

We investigated the epigenetic disparities in cuproptosis phenotypes, including SNV, CNV, and mutation signatures. Cluster 1 had a significantly higher TMB than Cluster 2 (Figure S7D), confirming the results of the immune infiltration analysis. Based on a previous study, we speculated that there are four mutation signatures related to gliomas: SBS1 (age associated), SBS5, SBS6 (DNA mismatch repair associated), and SBS14. However, only SBS1 demonstrated significant differences, implying that age was strongly associated with cuproptosis (Figure S7E–H). Therefore, these mutation signatures can be used to explain the disruption of each cuproptosis molecule phenotype.^31^ Among the top 20 frequently mutated genes, Cluster 1 had more mutations in *TP53*, *TTN*, *EGFR*, *PTEN,* and *PIK3CA*; however, Cluster 2 had a relatively higher mutation frequency in *IDH1, ATRX, CIC*, and *SMARCA4* (Figure S7C, upper part). Moreover, CNV frequency decreased in Cluster 2 but significantly increased in Cluster 1 (Figure S7C, middle part). Analysis of the frequently altered chromosomes supported our conclusions (Figure S7A,B).

### 3.5 Signaling pathway differences in cuproptosis patterns

We investigated the mechanisms underlying the effects of cuproptosis on gliomas by exploring the functions of cuproptosis molecules in several cancer‐related signaling pathways using The Cancer Proteome Atlas (TCPA) datasets. Cuproptosis molecules activated several signaling pathways, including the cell cycle, receptor tyrosine kinase, and hormone AR, and inhibited epithelial–mesenchymal transition (EMT), apoptosis, and DNA damage response in pan‐cancer (Figure S8A). In gliomas, many cuproptosis‐related genes could activate the androgen receptor, cell cycle, DNA damage response, and PI3K/AKT signaling pathways and inhibit TSC/mTOR and EMT signals (Figure S8B). Next, we conducted GSVA‐enrichment experiments to analyze the cancer‐related signaling pathways between the two distinct cuproptosis patterns. The inflammation‐related signaling pathways “TGF BETA signaling,” “PI3K AKT MTOR signaling,” and “G2 checkpoint” were significantly activated in Cluster 1, suggesting that Cluster 1 was significantly associated with immune‐relevant signatures. However, Cluster 2 performed better in “KRAS signaling” (Figure S8C). Additionally, the biological behaviors of the KEGG pathway enrichment patterns were analyzed. “Neuroactive ligand‐receptor interaction” upregulation was observed in Cluster 2, and cancer‐related pathways comprising “mismatch repair,” “DNA replication,” “homologous recombination,” and “cell cycle” were activated in Cluster 1 (Figure S8C). Referring to the published signature,^32^ we compared the enrichment scores of 10 carcinogen‐signaling pathways between both patterns. Signaling pathways, including TP53, TGF, RAS, PI3K, NRF2, and Hippo, scored higher in Cluster 1; however, the Wnt and Notch pathways were prevalent in Cluster 2 (Figure S8D). Furthermore, we investigated the relationship between the PI3K/AKT/mTOR signaling pathway and these two modalities in detail. PI3KRA (*p* < 0.001), AKT1 (*p* < 0.001), MTOR (*p* < 0.001), and CDK2 (*p* < 0.001) had higher expression levels in Cluster 1, suggesting that high levels of cuproptosis achieve immunosuppression by activating PI3K/AKT/mTOR signaling, promoting immune escape in Cluster 1 (Figure S8E). These analyses further confirmed that cuproptosis regulates the immune microenvironment and promotes immune escape in patients with glioma through multiple signaling pathways. This reveals the potential of cuproptosis molecules as immunotherapeutic targets.

### 3.6 FDX1 is associated with tumor progression in gliomas

**FIGURE 3:**
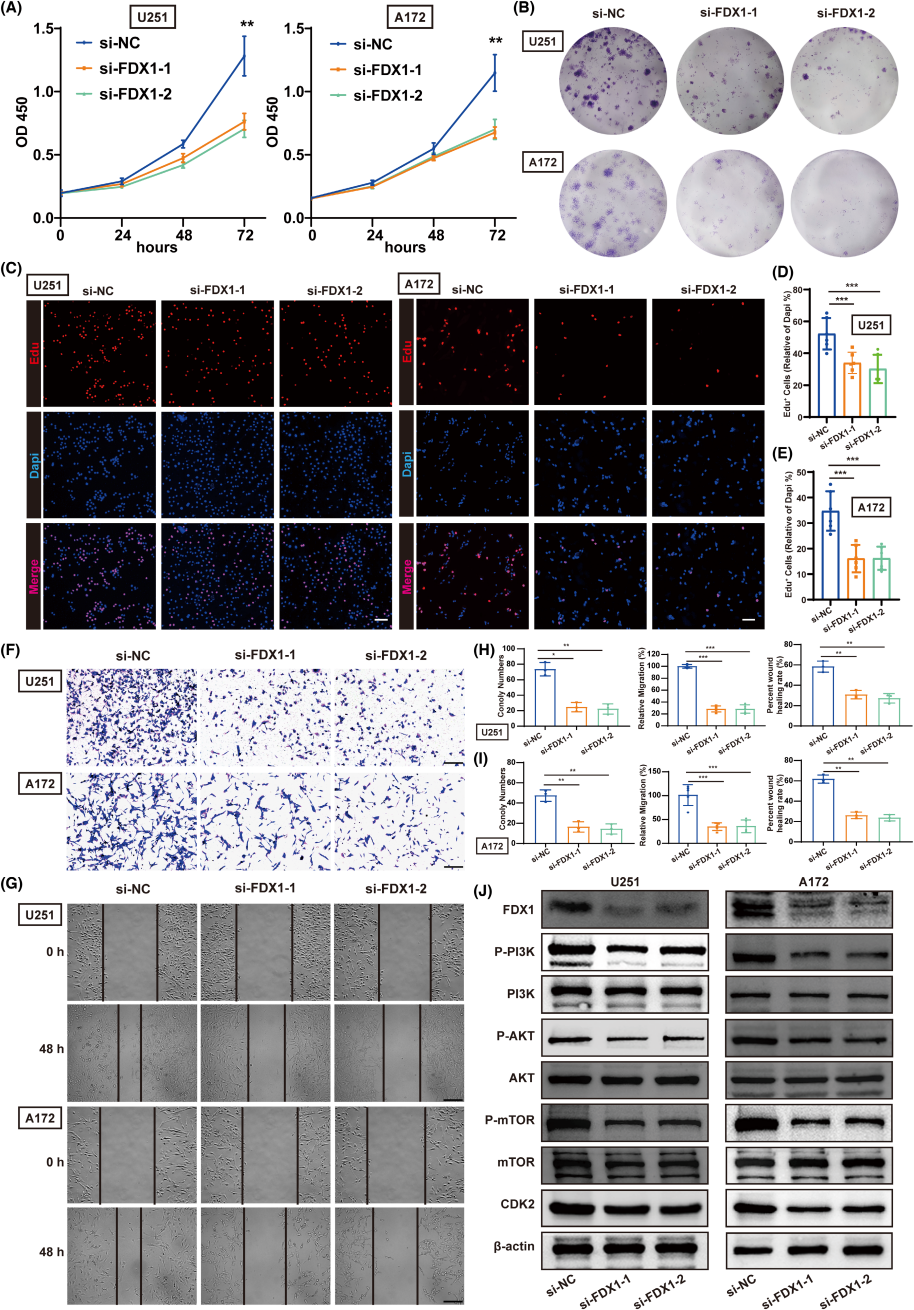
Low expression of FDX1 inhibited the proliferation and migration of glioma cells in vitro. (A) The CCK8 assay detected cell viability after decreased FDX1 expression in U251 and A172 cells (*n* = 6). (B) Knockdown of FDX1 significantly reduced the number of clones in U251 and A172 cells (*n* = 3). (C) The Edu assay detected cell proliferation after decreased FDX1 expression in U251 and A172 cells (*n* = 6, scale bars: 50 μm). (D, E) Quantification results of EdU^+^ cell rates in U251 (D) and A172 (E) cells. (F) The transwell assay detected the migration ability of U251 and A172 cells after decreased FDX1 expression (*n* = 5, scale bars: 400 μm). (G) The cell scratch assay detected the proliferation of U251 and A172 cells after decreased FDX1 expression (*n* = 3, scale bars: 400 μm). (H, I) Quantification results of numbers of clones in cell colony formation experiments, relative migration rates in transwell assay, and wound healing rates in cell scratch assay of U251 (H) and A172 (I) cells. The asterisks represented the statistical *p*‐value (one‐way ANOVA test: * *p* < 0.05; ** *p* < 0.01, *** *p* < 0.001). (J) Representative western blots examined the expression of FDX1, P‐PI3K, PI3K, P‐AKT, AKT, P‐mTOR, mTOR, and CDK2 protein levels after the downregulation of FDX1 of U251 and A172 cells.

Based on the results in Section 3.5, cuproptosis may be a glioma risk factor associated with the PI3K/AKT/mTOR pathway. Therefore, we focused on investigating the mechanism of action of *FDX1*, a hub regulator of cuproptosis, in vitro. Using univariate Cox analysis, we previously validated that *FDX1* was a risk factor for gliomas (Figure S2H). Two small interfering RNAs were used to downregulate *FDX1* expression in U251 and A172 cell lines. After transfection for 48 hours, western blotting demonstrated that *FDX1* was successfully knocked down (Figure 3J). Moreover, *FDX1* downregulation reduced cell viability (Figure 3A). Cell colony formation experiments showed that *FDX1* knockdown greatly reduced the number of colonies in U251 and A172 cells (Figure 3B). EdU assay demonstrates that the knockdown of FDX1 reduces cell proliferation in U251 and A172 cell lines (Figure 3C). Additionally, transwell and cell scratch assays demonstrated that *FDX1* knockdown significantly attenuated the migration ability of glioma cells (Figure 3F–I). Furthermore, western blotting showed that *FDX1* downregulation decreased P‐PI3K, P‐KAT, P‐mTOR, and CDK2 expressions but did not affect the overall PI3K, AKT, and mTOR protein expression (Figure 3J **; Figure S9 A, B**). These results demonstrate that *FDX1* promotes glioma proliferation and migration, which might be related to the PI3K/AKT/mTOR pathway.

### 3.7 Integrated construction of the CuproScore

**FIGURE 4:**
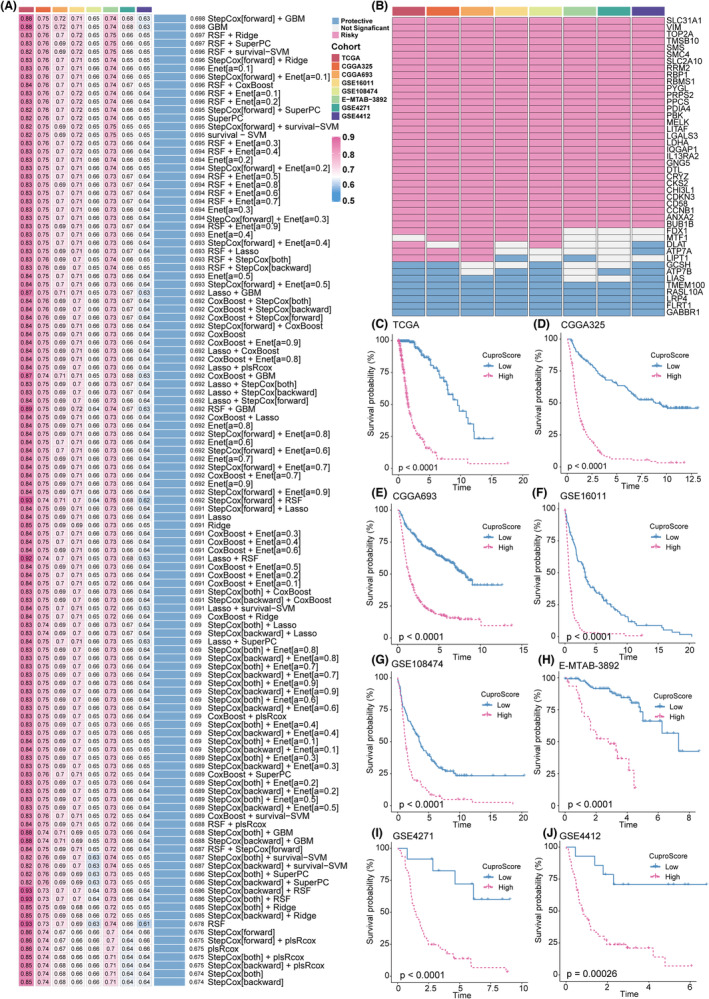
Construction of a machine learning–based signature. (A) The C‐index of 117 machine learning algorithms in eight validation cohorts. (B) This heatmap demonstrates the prognostic value of the 35 prognostic cuproptosis‐related DEGs specific to gliomas and nine stable prognostically significant cuproptosis genes in eight cohorts. (C–J) Kaplan–Meier curves of OS according to the CuproScore in TCGA gliomas (log‐rank test: *p* < 0.0001) (B), CGGA325 (log‐rank test: *p* < 0.0001) (C), CGGA693 (log‐rank test: *p* < 0.0001) (D), GSE16011 (log‐rank test: *p* < 0.0001) (E), GSE108474 (log‐rank test: *p* < 0.0001) (F), E‐MTAB‐3892 (log‐rank test: *p* < 0.0001) (G), GSE4271 (log‐rank test: *p* < 0.0001) (H), and GSE4412 (log‐rank test: *p* = 0.00026) (I)

To mine for cuproptosis‐related genes specific to gliomas, we screened out the differentially expressed genes (DEGs) between Cluster 1 and Cluster 2 with adj. *p* < 0.05 and |logFC| > 0.5 as cutoffs in all cohorts. The DEGs upregulated or downregulated in no less than six cohorts were defined as cuproptosis‐related genes specific to gliomas for further integrated analysis. Univariate regression analysis identified 35 prognostic cuproptosis‐related DEGs specific to gliomas from the intersection genes of the 8 cohorts (Figure 4B). Subsequently, a machine learning integrative procedure was performed on 35 cuproptosis‐related DEGs and 9 stable prognostically significant cuproptosis genes to construct a cuproptosis‐related signature, CuproScore. In the TCGA‐glioma cohort, we fitted 117 algorithm combinations based on the 10‐fold cross‐validation framework and calculated the C‐index of every combination in the remaining seven validation cohorts to assess the predictive power of all models. A model was evaluated based on whether it performed consistently across different cohorts. In this case, we selected the model with the highest average C‐index among the seven training cohorts as optimal. The combination of StepCox (forward) and gradient boosting machine (GBM) with the highest average C‐index (0.698) was considered the optimal model (Figure 4A and Table S2). Therefore, we calculated the CuproScore for each sample in all eight cohorts by the levels of the 44 cuproptosis‐related genes. The heatmap demonstrated CuproScore could significantly distinguish different cuproptosis statuses. The cuproptosis molecules in the high CuproScore group showed significantly higher expression levels in almost all eight cohorts and meta‐cohort (Figure S10A).

### 3.8 Consistent prognostic value of CuproScore

All the patients were dichotomized into high and low CuproScore groups. Kaplan–Meier survival analysis showed that the mortality rate in the high CuproScore group was significantly higher than that in the low CuproScore group in the training cohort of TCGA gliomas (*n* = 628, *p* < 0.0001). Similar result was observed in the validation cohorts CGGA325 (*n* = 309, *p* < 0.0001), CGGA693 (*n* = 657, *p* < 0.0001), GSE16011(*n* = 264, *p* < 0.0001), GSE108474 (*n* = 245, *p* < 0.0001), E‐MTAB‐3892(*n* = 151, *p* < 0.0001), GSE4271 (*n* = 77, *p* < 0.0001), and GSE4412 (*n* = 85, *p* = 0.0026) (Figures 4C–J).

Furthermore, multivariate Cox regression analysis was conducted to assess the independence of the CuproScore model from common clinical traits and molecular features, including grade, gender, age, 1p/19q co‐deletion, and IDH status. In TCGA gliomas, CGGA325, CGGA693, GSE16011, GSE108474, and GSE4271, CuproScore remained statistically significant after adjusting for these clinical and molecular variables (*p* < 0.05), suggesting its role as a significant independent risk factor for gliomas (Figure S11 A–E,G). However, given the small sample sizes of E‐MTAB‐3892 (*p* = 0.073) and GSE4412 (*p* = 0.389), CuproScore was not statistically significant (Figure S11F,H**)**. The calibration curves further proved the predictive accuracy of the CuproScore signature (Figure S11I–P).

### 3.9 Robust predictive performance of CuproScore

**FIGURE 5:**
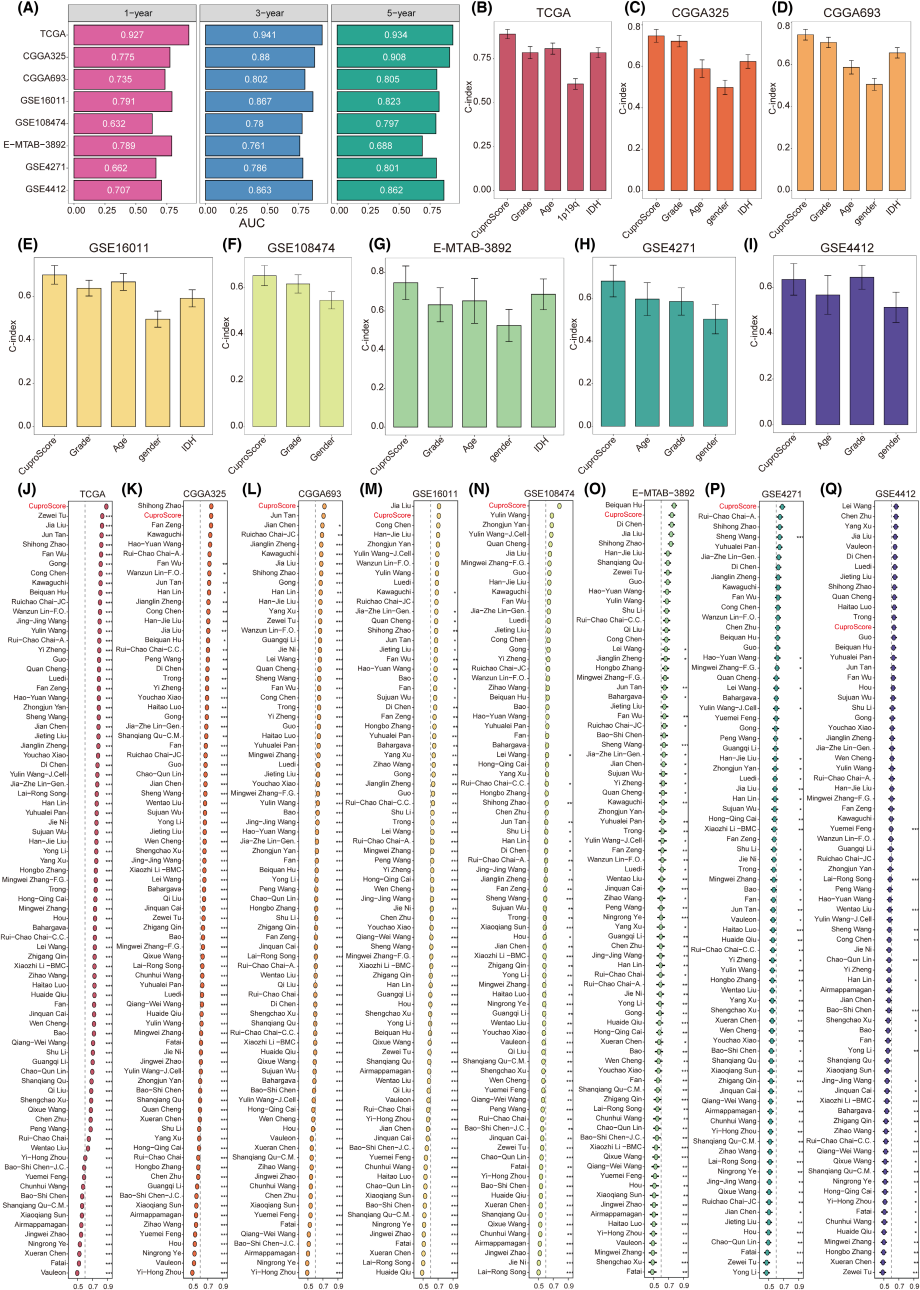
Comparison between the CuproScore and the other 80 signatures in Gliomas. (A) Time‐dependent ROC analysis for predicting OS at 1, 3, and 5 years in TCGA gliomas (*n* = 628), CGGA325 (*n* = 309), CGGA693 (*n* = 657), GSE16011 (*n* = 264), GSE108474 (*n* = 245), E‐MTAB‐3892 (*n* = 151), GSE4271 (*n* = 77), and GSE48276 (n = 85). (B‐I) The performance of the CuproScore was compared with other clinical traits in predicting prognosis. Statistic tests: two‐sided *z*‐score test. (J–Q) C‐indices of CuproScore and 80 published signatures in TCGA gliomas, CGGA325, CGGA693, GSE16011, GSE108474, E‐MTAB‐3892, GSE4271, and GSE4412. *Z*‐score test: *p < 0.05, **p < 0.01, ***p < 0.001, ****p < 0.0001.

We plotted receiver operating characteristic (ROC) curves to analyze CuproScore's discrimination effect. The areas under the ROC curve of 1‐, 3‐, and 5‐year OS were 0.927, 0.941, and 0.934, respectively, in TCGA gliomas, indicating CuproScore's superior performance in the training cohort. Similarly, excellent results were observed in the seven testing cohorts, including 0.775, 0.88, and 0.908 in CGGA325; 0.735, 0.802, and 0.805 in CGGA693; 0.791, 0.867, and 0.823 in GSE16011; 0.632, 0.780, and 0.797 in GSE108474; 0.789, 0.761, and 0.688 in E‐MTAB‐3892; 0.662, 0.786, and 0.801 in GSE4271; and 0.707, 0.863, and 0.862 in GSE4412, respectively (Figure 5A and Table S3). These results suggest that the CuproScore model's performance is stable and robust in multiple independent glioma cohorts.

Clinicians usually apply clinical and molecular features, such as grade, age, 1p/19q co‐deletion, IDH status, and gender, for treatment and prognostic evaluations. Therefore, we compared CuproScore's predictive accuracy with typical clinical and molecular features to predict the risk of death in patients with glioma by calculating the C‐index. CuproScore was significantly more accurate in the eight cohorts than in these variables (Figure 5B–I). Furthermore, we applied decision analysis to all cohorts and found that CuproScore provided the best net benefit (Figure S13B‐I). These results suggest that CuproScore might be a prospective alternative biomarker for predicting survival risk in clinical management.

### 3.10 Comparison of prognostic signatures in gliomas

Glioma treatment has become increasingly precise with the rapid development of high‐throughput sequencing. Numerous prognostic signatures of gliomas have recently been developed based on various machine learning algorithms. For a comprehensive comparison of CuproScore's performance with that of other signatures, we systematically retrieved mRNA signatures from glioma studies conducted over the past decade. Overall, we retrieved 80 mRNA signatures (Table S4).^33^ These 80 signatures are associated with different biological features, including aging, N6‐methyladenosine, glycolysis, ferroptosis, pyroptosis, immunotherapy response, stemness, and epigenetics. Next, we compared the predictive power of CuproScore and the other 80 signatures by calculating the C‐index in the eight cohorts. CuproScore ranked first in TCGA gliomas, CGGA693, GSE108474, and GSE4271, second in CGGA325, GSE16011, and E‐MTAB‐3892. Furthermore, our CuproScore model demonstrated distinctively superior accuracy than the other models in almost all cohorts (ranked in the top two of seven cohorts), revealing its robustness CuproScore (Figures 5J–Q). Some models exhibited superior performance in a few datasets but were weak in other external cohorts. For example, the signature reported by Beiquan Hu performed best in E‐MTAB‐3892 better than CuproScore; however, its performance in other cohorts was relatively poor, and its C‐index in GSE16011 was less than 0.6. Overall, models such as those derived by overfitting may be ineffective for generalization. These results demonstrate our model's superior stability and better extrapolation potential than others.

### 3.11 Immune characteristics related to CuproScore

We assessed the role of CuproScore in the TIME of gliomas by exploring the relationship between CuproScore and immune cell infiltration. Based on the ssGSEA algorithm, CuproScore was positively correlated with almost all tumor‐infiltrating immune cells in the TCGA cohort (Figure S12A). Additionally, we investigated the immune relevance of CuproScore separately in the meta‐GEO and CGGA cohorts (Figure S12B,C). As expected, analyses of immune cell infiltration and immune‐related pathways revealed positive correlations, indicating that the high CuproScore group was characterized by high immune cell infiltration and upregulation of immune‐related pathways, presenting an inflamed TIME phenotype. Moreover, we constructed heatmaps to validate the results using the TIMER, CIBERSORT, ESTIMATE, QUANTISEQ, MCP‐counter, XCELL, and EPIC algorithms (Figure S13A). Although the high CuproScore group had more abundant immune infiltration than the low CuproScore group, numerous immunosuppressive cells, such as macrophages, Tregs, monocytes, and MDSCs, played significant roles, possibly contributing to tumor immune escape. Next, we investigated the potential role of CuproScore in the anti‐tumor immune process and found that the high CuproScore group was positively correlated with all immune cycle stages (Figures S12A,D). Consistent with the above results, ESTIMATE analysis showed that the high CuproScore group had high stromal and immune scores and low tumor purity (Figures S12E–G). Moreover, several immune checkpoints were overexpressed in the high CuproScore group, including CD274, PDCD1, CTLA4, PDCDLG2, HAVCR2, and LG3 (Figure S12A). Notably, the high CuproScore group had significantly higher TIDE, exclusion, TMB, and CTA scores than the low CuproScore group. Moreover, patients with a low CuproScore had high MSI scores (Figures 12H‐L**)**, suggesting that patients with glioma with weak immune checkpoint expression and low TMB might benefit from ICI.

### 3.12 Mutation status in the low and high CuproScore groups

Somatic mutations in patients with glioma in the TCGA cohort were analyzed to investigate CuproScore‐related mechanisms. More mutations were found in the high CuproScore group than in the low CuproScore group (*R* = 0.46, *p* < 0.001; Figure S14A), including synonymous (*R* = 0.44, *p <* 0.001; Figure S14B) and non‐synonymous mutations (*R* = 0.46, *p* < 0.001; Figure S14C). Moreover, a forest plot revealed that the low CuproScore group had a considerably greater mutation frequency of *IDH1*, *CIC*, *ATRX*, *FUBP1*, and *TP53*; however, the high CuproScore group had more mutations of *TTN*, *NF1*, *PTEN*, and *EGFR* (Figure S14D). Additionally, we observed substantial co‐occurrence among these genes (Figure S14E). Lastly, CuproScore was positively correlated with the mutation frequency of the immunotherapy‐related gene *PTEN* on studying the effect of significant genetic mutation variations, suggesting that the low CuproScore group might benefit more from ICI than the high CuproScore group (Figure S14F).

### 3.13 CuproScore predicts the response of gliomas to immunotherapy

**FIGURE 6:**
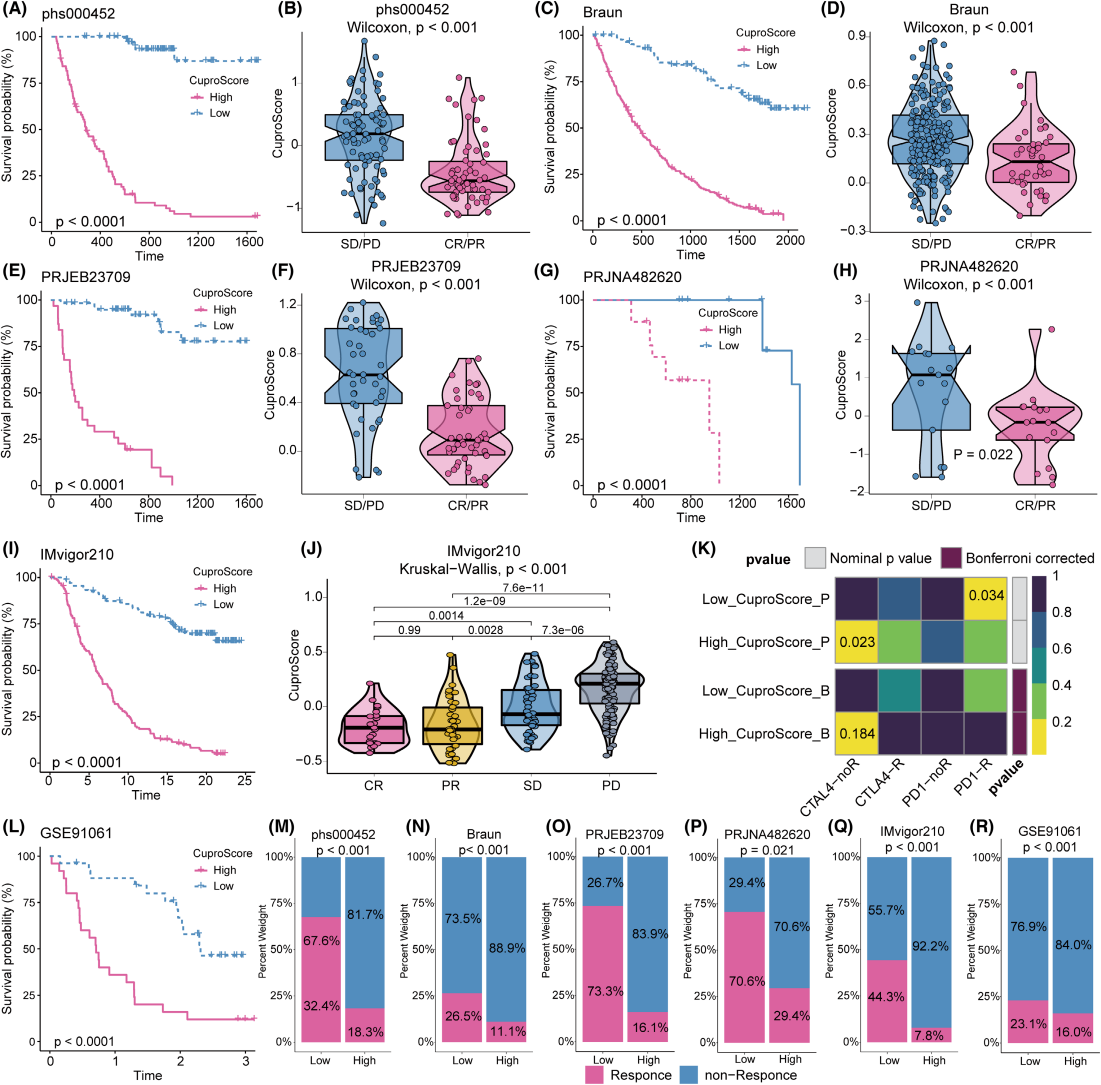
CuproScore Predicts the Response of Gliomas to Immunotherapy. (A) The Kaplan–Meier curve showed a significant difference in survival rate between the high and low CuproScore groups in the phs000452 cohort. (B) The Wilcoxon rank‐sum test of CuproScore variation in anti‐PD‐L1 responsiveness in the phs000452 cohort. (C) The Kaplan–Meier curve showed a significant difference in survival rate between the high and low CuproScore groups in the Braun cohort. (D) The Wilcoxon rank‐sum test of CuproScore variation in anti‐PD‐L1 responsiveness in the Braun cohort. (E) The Kaplan–Meier curve showed a significant difference in survival rate between the high and low CuproScore groups in the PRJEB23709 cohort. (F) The Wilcoxon rank‐sum test of CuproScore variation in anti‐PD‐L1 responsiveness in the PRJEB23709 cohort. (G) The Kaplan–Meier curve showed a significant difference in survival rate between the high and low CuproScore groups in the PRJNA482620 cohort. (H) The Wilcoxon rank‐sum test of CuproScore variation in anti‐PD‐L1 responsiveness in the PRJNA482620 cohort. (I) The Kaplan–Meier curve showed a significant difference in survival rate between the high and low CuproScore groups in the IMvigor210 cohort. (J) The Kruskal–Wallis test of CuproScore variation in anti‐PD‐L1 responsiveness in the IMvigor210 cohort. (K) The submap algorithm predicts the probability of anti‐PD1 and anti‐CTLA4 immunotherapy response in high and low CuproScore groups. (L) The Kaplan–Meier curve showed a significant difference in survival rate between the high and low CuproScore groups in the GSE91061 cohort. (M‐R) The stacked histogram shows the difference in immunotherapy responsiveness between the high and low CuproScore groups in phs000452 (M), Braun (N), PRJEB23709 (O), PRJNA482620 (P), IMvigor210 (Q), and GSE91061 (R).

As previously demonstrated, the low CuproScore group had low TIDE scores, high MSI scores, high CTA score, and less *PTEN* mutations, implying that immunotherapy might be more beneficial for patients in this group (Figure S12I‐L; Figure S14D, F). Next, we confirmed CuproScore's predictive value in the immunotherapy cohort. The phs000452 cohort was divided into two CuproScore groups. Patients with low CuproScores had better prognoses after PD‐L1 treatment (Figure 6A). As expected, patients with metastatic melanoma with low CuproScores were more likely to respond to anti‐PD‐L1 immunotherapy (Figures 6B,M). In the Braun cohort, patients with low CuproScores had better prognoses after PD‐L1 treatment (Figure 6C). As expected, patients with renal cell carcinoma with low CuproScores were more likely to respond to anti‐PD‐L1 immunotherapy (Figure 6D,N). In the PRJEB23709 cohort, patients with low CuproScores had better prognoses after PD‐L1 treatment (Figure 6E). As expected, patients with metastatic melanoma with low CuproScores were more likely to respond to anti‐PD‐L1 immunotherapy (Figure 6F, O). In the GSE91061 cohort, patients with low CuproScores had better prognoses after PD‐L1 treatment (Figure 6L, R). Notably, in an immunotherapy dataset of patients with glioblastoma, PRJNA482620, survival analyses demonstrated a significant prognostic advantage in the low CuproScore group after anti‐PD‐1 immunotherapy (Figure 6G). As expected, patients with glioblastoma with low CuproScores were more likely to respond to anti‐PD‐L1 immunotherapy (Figure 6H, P). Additionally, patients with low CuproScores in the IMivgor210 cohort had better prognoses after PD‐L1 treatment (Figure 6I). As expected, patients with urothelial carcinoma with low CuproScores were more likely to respond to anti‐PD‐L1 immunotherapy (Figures 6J, Q).

Furthermore, we employed a submap algorithm to calculate CuproScore's ability to forecast the impact of immunotherapy. The results confirmed that patients in the low CuproScore group might benefit more from anti‐PD1 therapies (Figure 6K). Overall, our CuproScore model has the potential to be an effective tool for evaluating prognosis and clinical response to anti‐PD1 and anti‐CTLA4 immunotherapy. Therefore, CuproScore is essential for establishing a new glioma treatment strategy.

### 3.14 CuproScore predicts sensitivity of gliomas to chemotherapeutic response analysis

We investigated the relationship between cuproptosis molecules and the chemotherapeutic agents used to treat gliomas. According to the GDSC drug response data, drugs and some genes, such as *SLC31A1* and *GLS*, can interact synergistically; however, *ATP7A*, *LIAS*, *FDX1*, *DLAT*, *MTF1*, and *PDHB* interacted antagonistically with drugs (Figure S15A). Notably, *SLC31A1* exhibited a substantial synergistic interaction with Navitoclax (apoptosis regulator). Moreover, *LIAS* showed strong negative interaction with JW‐7‐52‐1 (PI3K/MTOR signaling inhibitor), KIN001‐260 (Bayer IKKb inhibitor), and YM201636 (PI3K/MTOR signaling inhibitor). Furthermore, we explored the cuproptosis genes and drug sensitivity using the Clinical Trials Reporting Program database (Figure S15B). Given the vital role of cuproptosis molecules in chemotherapy, we further analyzed CuproScore's potential to guide the clinical selection of chemotherapeutic agents for glioma. Differences in the chemotherapeutic agents' half‐maximal inhibitory concentration (IC50) between patients with high and low CuproScores were predicted using ridge regression. Patients with lower CuproScore had lower IC50 for several chemotherapy drugs, including Axitinib, ATRA, Bosutinib, Lenalidomide, IPA.3, Gefitinib, Elesclomol, Nutlin.3a, Nilotinib, Vorinostat, BAY.61.3606, and EHT.1864 (Figure S15C), suggesting that chemotherapy was a promising option for the low CuproScore group. Notably, Elesclomol could effectively induce cuproptosis.

## 4 DISCUSSION

Glioma is a prevalent type of intracranial tumor with a poor prognosis.^34^, ^35^ Owing to the complex pathogenesis of gliomas and the strong proliferative capacity of the cells, treating patients with gliomas is challenging.^36^, ^37^ Patients have high recurrence rates and poor prognoses after surgical treatment, with survival time extended by only a few months.^38^, ^39^ Similarly, radiotherapy and chemotherapy have low success rates.^40^, ^41^ Furthermore, immunotherapy is a novel therapeutic strategy for treating patients with gliomas. However, it is ineffective for most patients with glioma, particularly those with glioblastoma, with only 10% benefitting.^42^ Therefore, identifying novel therapeutic targets for glioma immunotherapy is vital.^43^

Cell death prevents tumor over‐proliferation and maintains homeostasis.^44^, ^45^ Numerous studies have shown that metabolism is closely associated with cell death in cancer.^46^ As an important trace element in humans, copper ions are involved in metabolic processes, such as mitochondrial respiration and energy metabolism, by binding to various proteins or enzymes as cofactors or structural components.^47^, ^48^ An imbalance in copper ions can cause aberrant autophagy and oxidative stress, inducing various copper‐ion‐related diseases. Golub et al. recently proposed that intracellular copper overload causes the accumulation of lipid‐acylated proteins in the mitochondria and the loss of iron–sulfur cluster proteins, resulting in cell death. This copper‐dependent cell death is termed cuproptosis.^13^ Numerous studies have shown that copper is essential to tumor growth and immunity.^49^, ^50^ Cancer cells have higher copper requirements than normal cells.^51^, ^52^, ^53^ Therefore, blocking copper‐ion transport can lead to oxidative stress, inhibiting tumor cell proliferation.^18^ Moreover, reducing copper levels in vivo may reduce tumor vascular winds and inhibit tumor growth.^54^ Although prognostic models based on cuproptosis in gliomas have been established,^24^, ^25^ the effects of cuproptosis on signaling pathways and the tumor microenvironment and its prediction performance for immunotherapy in gliomas remain poorly understood.

This study demonstrated the genetic and transcriptomic heterogeneity of 16 cuproptosis‐related molecules in 32 cancer species. The imbalance of cuproptosis molecule expression was positively correlated with CNV. Moreover, we used unsupervised clustering in the eight independent glioma cohorts, meta‐cohort, LGG cohort, and glioblastoma cohort, respectively, to classify patients with gliomas into two cuproptosis molecule phenotypes. We found significant differences in the clusters' genetic characteristics and immune infiltration.

Cluster 1 was characterized by higher expression of cuproptosis genes and higher enrichment of almost all immune cells than Cluster 2, presenting an inflamed TIME phenotype. Additionally, Cluster 2 was characterized by lower immune cell infiltration and high tumor purity, presenting an immune desert phenotype. Notably, a worse prognosis was observed in Cluster 1 patients, and cluster 2 was identified as an independent prognostic factor. These contradictory results might be related to immune escape due to the unique immune microenvironment of Cluster 1. We first investigated the extrinsic immune escape mechanism based on immunoediting theory from previous studies.^55^ Cluster 1 was densely infiltrated by CD8^+^ T cells, but M2 macrophages, Treg cells, and MDSCs were activated, too. Glioma tissues from our hospital were used to confirm the positive correlation between the level of cuproptosis and macrophages. These immune cells had the highest density in gliomas and possessed immunosuppressive cytokines and chemokines, including CXCL12, CXCL10, CCR5, CCR10, CCL5, and CCR2, to exert their immunosuppressive effects. Moreover, macrophages and MDSCs inhibited cytotoxic responses mediated by NK cells and blocked CD8^+^ T cell activation.^6^ Furthermore, TGFβ and PI3K/AKT pathways were activated in Cluster 1, which might lead to T cell dysfunction,^56^ consistent with high TIDE and exclusion scores of Cluster 1. Meanwhile, Cluster 1 had a high CAF enrichment, indicating a high degree of fibrosis. These results demonstrate that immunosuppressive cells, high concentrations of immunosuppressive factors, high levels of fibrosis, and activation of immunosuppressive signaling pathways in Cluster 1 induced T cell dysfunction, triggering extrinsic immune escape.

Next, we analyzed the potential intrinsic immune escape mechanisms in Cluster 1. We discovered many immune escape‐related gene mutations in Cluster 1, such as in *PTEN*.^57^ Moreover, Cluster 1 patients had higher levels of immune checkpoints and TMB than Cluster 2 patients, implying that Cluster 1 was more immunogenic and capable of producing tumor neoantigens. However, higher immune checkpoints hinder antigen presentation, constituting the intrinsic immune escape mechanism of Cluster 1. Therefore, although Cluster 1 patients with higher cuproptosis levels had a hotter tumor microenvironment, tumor suppressor components had the highest density in gliomas. Consequently, the activated anti‐tumor component is insufficient to initiate an anti‐tumor immune response by overcoming the immunosuppressive component, explaining why Cluster 1 patients had a stronger immune escape capability and a more immunosuppressive environment than Cluster 2 patients. Therefore, we hypothesized that cuproptosis might be an essential component of the immune escape mechanism of gliomas, providing a novel target for immunotherapy of gliomas. Moreover, to demonstrate the specific role of cuproptosis in gliomas, we selected *FDX1*, a key regulator of cuproptosis, for cellular experiments. *FDX1* promoted glioma cell proliferation and migration, possibly through the PI3K/AKT/mTOR pathway.

Considering the vital role of cuproptosis in gliomas, developing a cuproptosis‐based signature is crucial to accurately evaluate prognosis and predict the gliomas' immunotherapy response. However, in existing studies, researchers mostly selected modeling algorithms relying largely on their preferences and biases. Therefore, we collected 10 classical algorithms and combined them into 117 combinations. Next, we constructed a machine learning–based cuproptosis‐related signature, CuproScore, with the best performance among the 117 models. Notably, biomedical model development with artificial intelligence and machine learning is commonly hampered by overfitting, where several models often fit well in the training cohort but poorly in other external validation cohorts. In this case, we performed all models in one training cohort and seven validation cohorts and screened the most valuable signature with the highest average C‐index.

Moreover, compared to 80 published glioma signatures, CuproScore showed superior accuracy, demonstrating its robustness. Additionally, CuproScore was highly accurate in assessing patient survival outcomes. The poor prognosis of the high CuproScore group may be related to the activation of anti‐immune components with high tumor immune escape. Furthermore, CuproScore was positively correlated with the level of tumor immune infiltration in gliomas. Additionally, mutations generate tumor neoantigens, and a high TMB increases the number of tumor immunogenic neoantigens.^58^, ^59^ Consequently, immunotherapy may be more beneficial for patients with a high TMB. Nevertheless, many patients with high TMB do not benefit from immunotherapy.^60^ Similarly, the high CuproScore group with higher TMB in our study did not benefit from immunotherapy. Our findings demonstrate the limitations of TMB as a predictive biomarker, especially when used alone.^61^ Furthermore, when the immunotherapy response was evaluated using TIDE and MSI, the group with low CuproScore and TIDE scores and high MSI responded better to immunotherapy, possibly due to lower tumor immune escape from T cell exclusion.^62^ We validated these findings in other cancer cohorts treated with immunotherapy. Notably, in the immunotherapy glioma cohort, the low CuproScore group responded better. Moreover, drug sensitivity analysis showed that the low CuproScore group was more sensitive to more chemotherapeutic agents, proposing another treatment strategy. Notably, studies on the TIME of gliomas have demonstrated that immunosuppressive environments inside tumors underlie tumor resistance to immunotherapy. Additionally, in gliomas, macrophages are immunosuppressive and are associated with poor prognoses.^63^ These characteristics of gliomas necessitate developing immunotherapeutic strategies that differ from those for other solid tumors. Overall, these results suggest that CuproScore could be a promising tool for designing more effective glioma treatment strategies.

This study has some limitations. First, although we compiled 16 cuproptosis genes from the literature, newly discovered cuproptosis genes should be included in the model to optimize accuracy. Additionally, we used retrospective cohorts from public online databases; larger multicenter prospective clinical studies should be conducted to validate these findings. Lastly, to demonstrate the relevance of CuproScore in predicting response to immunotherapy, we need more indicators for validation and prospective cohorts of patients with gliomas receiving immunotherapy.

In conclusion, we described the TIME, mutation landscape, and altered signaling pathways between different cuproptosis molecular patterns, revealing unique immune escape mechanisms. Based on the multicenter integrative analysis and machine learning algorithms, we developed a stable and robust signature, CuproScore, for predicting the prognosis and immunotherapy response in gliomas. CuproScore is a promising tool for designing more effective glioma treatment strategies.

## AUTHOR CONTRIBUTIONS

Shi Feng and Hua Zhu analyzed the data. Xiaoxing Xiong, Lijuan Gu, and Ning Zou designed this study. Shi Feng and Yonggang Zhang wrote the article. Yonggang Zhang conducted cellular experiments. Zhi Zeng and Xu Zhang evaluated the immunohistochemical staining results. Daniel Smerin, Zhihong Jian, Yingze Ye, and Yina Li revised the manuscript.

## FUNDING INFORMATION

This work was supported by the Fundamental Research Funds for the Central Universities (2042022kf1216) to Xiaoxing Xiong, and the National Natural Science Foundation of China (Nos 82271370) to Lijuan Gu.

## CONFLICT OF INTEREST STATEMENT

All authors declare no conflict of interest.

## CONSENT FOR PUBLICATION

Every author approved the manuscript before submission for publication.

## Supporting information


Table S1

Table S2

Table S3

Table S4

Appendix S1

## Data Availability

The data that support the findings of this study are available from the corresponding author upon reasonable request.
