# Supplementary material for: Cuproptosis facilitates immune activation but promotes immune escape, and a machine learning–based cuproptosis‐related signature is identified for predicting prognosis and immunotherapy response of gliomas
Source: CNS Neurosci Ther. 2023 Jul 28;30(2):e14380. doi: 10.1111/cns.14380 (PMC10848101; doi:10.1111/cns.14380)
Supplement: Supplementary file 5 — Appendix S1 [file CNS-30-e14380-s004.docx]

**Supplementary methods**

**Acquisition and Preprocessing of Data**

All the data utilized in this study came from public databases. TOIL-processed transcriptome data for TCGA-LGG, TCGA-glioblastoma, and GTEx-brain were obtained from the UCSC database (https://xenabrowser.net/datapage/). We downloaded RNA sequencing data and corresponding clinicopathological information from the CGGA website (https://www.cgga.org.cn). The Gliovis website (http://gliovis.bioinfo.cnio.es/) was used to download the other five glioma cohorts, including GSE16011, GSE108474, GSE4271, GSE4412, and E-MTAB-3892. The RNA sequencing data was transformed into transcripts per kilobase million (TPM) format. The microarray data from the Affymetrix platform were normalized and corrected background by the "affy" R package. Clinicopathologic phenotypes corresponding to transcriptomic data were searched and retrieved from the cbioportal database (http://www.cbioportal.org/). A total of 2416 patients were included after excluding samples without survival information. We combined the eight cohorts into an integrated meta-cohort. Batch effects were corrected using the "sva" package in R software[1]. We extract the LGG cohort and glioblastoma cohort from the meta-cohort. TCGA-LGG and TCGA-glioblastoma genomic mutation data, including Single Nucleotide Variation (SNV) and Copy Number Variation (CNV), were obtained from the UCSC database and analyzed using the "maftools" R package for SNV data. Amplification and deletion of CNV were detected using GISTIC2.0. We obtained RPPA proteomics data and calculated activation and inhibition scores for ten broadly acknowledged cancer-related pathways in 32 cancers via the TCPA database. On the GSCAlite website (http://bioinfo.life.hust.edu.cn/web/GSCALite/), partial TCGA pan-cancer analysis and RPPA data analysis were implemented[2].

**Unsupervised Clustering of Cuproptosis Genes**

Sixteen cuproptosis genes were obtained from previous research[3-5]. We identified nine stable prognostic genes by Univariate cox analysis in eight cohorts. The meta-cohort, LGG cohort, glioblastoma cohort, and all eight cohorts were clustered unsupervised using the unsupervised average linkage K-means clustering analysis [6, 7] and repeated 1000 times to ensure classification stability[8]. We then used principal component analysis (PCA) to validate the clustering based on the expression profiles of the above genes.

**Evaluation of tumor immune microenvironment**

We evaluated tumor purity, immune score, and stromal score using the "ESTIMATE" algorithm[9]. We also calculated the level of immune cell infiltration in gliomas using the ssGSEA algorithm[10], CIBERSORT algorithm[11], XCELL algorithm[12], TIMER algorithm[13], QUANTISEQ algorithm[14] and MCPCOUNTER algorithm[15]. We obtained a list of 100 immunomodulatory genes, including chemokine receptors, interleukins, and interferons, from existing studies[10, 16]. We analyzed the tumor immune dysfunction and exclusion (TIDE) score on the online website (http://tide.dfci.harvard.edu/). The TIDE score correlates with the degree of T cell dysfunction and immune factor exclusion of T cells in malignancies with high cytotoxic T cells (CTL)[17]. Lower TIDE scores indicate sensitivity to immunotherapy. We downloaded the "TME-associated signature" gene sets using the "IBOR" R package[18] and examined the scores of these gene sets in gliomas using the ssGSEA algorithm. We downloaded gene data from the website tracking tumor immunity (http://biocc.hrbmu.edu.cn/TIP/index.jsp) for seven steps of the cancer immune cycle[19] and quantified them by the ssGSEA algorithm.

**Analysis of pathway enrichment**

First, we investigated the correlation between cuproptosis molecules and cancer-related pathways in 32 cancers. The pathway score is calculated by taking the difference between the protein levels of all positive and negative regulatory components and adding them up. Patients were divided into two groups based on median protein levels of cuproptosis molecules[20]. When the high-expression group scored higher than the low-expression group, it was determined that the pathway was in an active condition[21]. To discover cancer pathway expression at the level of the transcriptome, we collected gene sets of the ten cancer-related pathways mentioned above from previous research and scored them using the ssGSEA algorithm. To investigate the biological differences between the two cuproptosis molecular patterns, we downloaded "h.all.v7.4.symbols" and "c2.cp.kegg.v7.4.symbols" from the MsigDB database (https://www.gsea-msigdb.org/gsea/msigdb) and applied the GSVA program[22].

**Development of the CuproScore Prognostic Model**

As previously reported[23-25], we converted gene expression profiles to z‐scores in all cohorts to enhance comparability between different cohorts. Cohorts with survival information, including TCGA-Gliomas, CGGA325, CGGA693, GSE16011, GSE108474, GSE4271, GSE4412, and -MTAB-3892 were utilized to develop CuproScores in the following steps:

(1) First, we determined the differentially expressed genes (DEGs) between the two cuproptosis clusters in the eight cohorts using the "limma" R package, with the filtering criteria for DEGs being adj. p<0.05 and abs(logFC)>0.5. The DEGs upregulated or downregulated in no less than six cohorts were defined as cuproptosis-related genes specific to gliomas for further integrated analysis. Afte using univariate COX regression, 35 DEGs were found prognostically significant in all cohorts. The 35 DEGs and nine stable prognostically significant cuproptosis genes were included to construct the model.

(2) Next, we applied ten classical machine learning algorithms, including random forest (RSF), elastic network (Enet), stepwise Cox, CoxBoost, partial least squares regression for Cox (plsRcox), supervised principal components (SuperPC), gradient boosting machine (GBM), survival support vector machine (Survival-SVM), Ridge, and least absolute shrinkage and selection operator (LASSO). Some algorithms could serve as feature selection tools, such as Lasso, stepwise Cox, CoxBoost, and RSF. Therefore, these algorithms were integrated into 117 combinations to fit a prognostic model. Tenfold cross-validation was used to screen for the most valuable signature with the highest C-idex.

(3) We took the TCGA-Gliomas cohort as the training set for 117 algorithms, while the other seven cohorts were considered as validation cohorts. We calculated the CuproScore for every cohort based on the model obtained in the training cohort.

(4) For each model, the C-index was calculated in all validation cohorts, and the model with the highest average C-index was considered optimal.

**The Prediction of immunotherapy Response**

We predicted the immunotherapy response of CuproScore by analyzing the expression of immune checkpoints, TMB, and TIDE score. Additionally, we utilized immunotherapy cohorts with comprehensive clinical information to predict immunotherapy response. Using the IMvigor210CoreBiologies R package; the transcriptome data, survival data, and immunotherapy effects of the IMvigor210 cohort were obtained[26]. After eliminating samples without survival time information, 298 samples remained. The expression data, clinical information, and immunotherapy results of the GSE91061[27], phs000452[28], Braun[29], PRJEB23709[30], and PRJNA482620[31] cohorts were downloaded from the TIGER website (http://tiger.canceromics.org/)[32]. And the transcriptome matrix was converted into the TPM format. These cohorts of patients were divided into four groups: progressive disease (PD), stable disease (SD), partial remission (PR), and complete remission (CR). We calculated the CuproScore of every patient in these cohorts to investigate the impact of the CuproScore on immunotherapy. Subclass Mapping (Submap) was utilized to determine correlations between high or low CuproScore groups and anti-PD-1 and CTAL4 immune checkpoint therapy[33, 34].

**The Prediction of Chemotherapeutic Sensitivity**

The correlation between cuproptosis molecules and drug sensitivity was predicted using the GDSC and CTRP datasets. We used the R package "PRRophetic" to predict the relationship between CuproScore and chemotherapy drug IC50. Some cuproptosis genes and drug correlations were achieved by GSCALite. (<http://bioinfo.life.hust.edu.cn/web/GSCALite/>).

**Cell Culture**

Human glioma cell line U87 was obtained from Huzhou Central Hospital. Cells were maintained in Dulbecco's Modified Eagle Medium (DMEM, Gibco, 11995040) containing 10% FBS and 1% penicillin-streptomycin at a cell incubator (37 °C, 5% CO2).

**siRNA transfection**

U251 and A172 cells were seeded in a 6-well plate. Then, U251 and A172 cells were transfected with si-FDX1-1 (sequence: 5′-CUAACAGACAGAUCACGGUTT-3′), si-FDX1-2 (sequence: 5′- GUGAUUCUCUGCUAGAUGUTT -3′), or negative control siRNA (si-NC, sequence: 5′- UUCUUCGAACGUGUCACGUTT -3′) using LipofectamineTM 2000 (Invitrogen, USA) reagent according to the protocol. 48 hours after transfection, Western blot analysis was performed to detect the knockdown efficiency.

**Cell viability assay**

48 hours after transfection, cells were harvested and seeded into 96 well plates at 2000 cells/well. Cell viability was accessed by a cell counting kit-8 (Beyotime, C0037) at 0, 24, 48, 72 hours according to the instruction manual.

**Cell lysis and immunoblotting**

U251 and A172 cells were lysed with cold RIPA buffer on ice. Total protein was quantified with BCA protein assay Kit (Tiangen). Equal amounts of protein samples were loaded and separated by SDS-PAGE electrophoresis and transferred to nitrocellulose membranes. Primary antibodies were incubated with the samples at 4℃, overnight. All antibodies were as follows: anti-IBA1 (ab283319, Abcam), anti-SLC31A1 (67221, Proteintech), anti-CD68 (25747, Proteintech), anti-FDX1 (12592, Proteintech), anti-P-PI3K (AF3242, Affinity), anti-PI3K (AF6241, Affinity), anti-P-AKT (AF0016, Affinity), anti-AKT (AF0836, Affinity), anti-P-mTOR (AF3308, Affinity), anti-mTOR (AF6308, Affinity), anti-CDK2 (AG1561, Beyotime), and anti-Tubulin (ab7291, Abcam). Horseradish peroxidase-conjugated goat anti-mouse immunoglobulin or goat anti-rabbit immunoglobulin (1:3000, Affinity) were used as the secondary antibody, which was incubated with the above mixture for another 2 hours at room temperature. Detection was performed on an Immobilon Western chemiluminescent HRP substrate kit (Millipore) according to the manufacturer’s instructions (Syngene). The densitometry data were analyzed with Image J software.

**Transwell assay**

U251 and A172 cells transfected with si-FDX1-1, si-FDX1-2, or si-NC were harvested, washed with PBS two times, and resuspended in DMEM. Then cells were seeded into the upper chamber of the 24-well compartments with an 8 μm pore size insert. 700 μl DMEM with 10% FBS was added in the lower chamber. 24 hours after incubation, cells on the surface of the upper chamber were carefully removed with a cotton swab. Cells on the surface of the bottom chamber were fixed with 4% PFA and stained with crystal violet. Images were then captured and analyzed.

**Wounding healing assay**

U251 and A172 cells were seeded into a 6-well plate and transfected with siRNA to knockdown FDX1. Cells were then scratched in each well with a pipette tip after they had grown to nearly 100% density. Images were captured at 0 and 48 hours after scratching. The wound healing rate was then analyzed by Image J software.

**Cell colony formation experiments**

Colony formation assays were conducted by planting 500 transfected cells in 6-well plates. After 14 days, the colonies were recorded and counted after the cells were fixed and stained.

**EdU assay**

48 hours after transfection, cells were harvested and seeded on coverslips. After 24 hours, cells were incubated with EdU (Beyotime, C0071S) for 2 hours at 37℃. Then, cells were fixed and stained to detect EdU.

**Immunohistochemical staining**

The FDX1, SLC31A1, IBA1, and CD68 expression in 56 clinical patients’ tissues was detected by immunohistochemistry (IHC). Paraffin sections immunohistochemistry was conducted as mentioned previously[35]. Protein expression was calculated as described previously[36]. Percentage of positive cells was graded as 0 (negative), 1 (up to 10%), 2 (11-50%), 3 (51-75%), or 4 (>75% positive cells), and staining intensity as 0 (no staining), 1 (weak), 2 (moderate), or 3 (strong). The final immunoreactive score was defined as the multiplication of both grading results (percentage of positive cells * staining intensity).

**Statistical analysis**

All statistical analyses were performed by R software (version 4.0.2). The Wilcoxon rank-sum test was used for the analysis of nonnormally distributed variables between the two groups. The t-test was employed for the statistics of normally distributed variables. Comparisons between three or more groups were conducted using one-way ANOVA and Kruskal-Wallis to test for normally or non-normally distributed variables. Categorical variables were analyzed using the Fisher test and the chi-square test. Correlations were calculated using the spearman and Pearson techniques. Using the Kaplan-Meier method and the Log-Rank test, the survival differences between groups were determined. For the calculation of risk ratios and confidence intervals, we used univariate and multivariate COX. Statistical significance was defined as *p* < 0.05.

**References:**

[1] B. Zhang, Q. Wu, B. Li, D. Wang, L. Wang, Y.L. Zhou, m(6)A regulator-mediated methylation modification patterns and tumor microenvironment infiltration characterization in gastric cancer, Mol Cancer, 19 (2020) 53.

[2] C.J. Liu, F.F. Hu, M.X. Xia, L. Han, Q. Zhang, A.Y. Guo, GSCALite: a web server for gene set cancer analysis, Bioinformatics, 34 (2018) 3771-3772.

[3] D. Tang, X. Chen, G. Kroemer, Cuproptosis: a copper-triggered modality of mitochondrial cell death, Cell Res, 32 (2022) 417-418.

[4] P. Tsvetkov, S. Coy, B. Petrova, M. Dreishpoon, A. Verma, M. Abdusamad, J. Rossen, L. Joesch-Cohen, R. Humeidi, R.D. Spangler, J.K. Eaton, E. Frenkel, M. Kocak, S.M. Corsello, S. Lutsenko, N. Kanarek, S. Santagata, T.R. Golub, Copper induces cell death by targeting lipoylated TCA cycle proteins, Science, 375 (2022) 1254-1261.

[5] Y. Wang, L. Zhang, F. Zhou, Cuproptosis: a new form of programmed cell death, Cell Mol Immunol, 19 (2022) 867-868.

[6] M. Seiler, C.C. Huang, S. Szalma, G. Bhanot, ConsensusCluster: a software tool for unsupervised cluster discovery in numerical data, OMICS, 14 (2010) 109-113.

[7] X. Qin, J. Li, W. Hu, J. Yang, Machine Learning K-Means Clustering Algorithm for Interpolative Separable Density Fitting to Accelerate Hybrid Functional Calculations with Numerical Atomic Orbitals, J Phys Chem A, 124 (2020) 10066-10074.

[8] M.D. Wilkerson, D.N. Hayes, ConsensusClusterPlus: a class discovery tool with confidence assessments and item tracking, Bioinformatics, 26 (2010) 1572-1573.

[9] K. Yoshihara, M. Shahmoradgoli, E. Martinez, R. Vegesna, H. Kim, W. Torres-Garcia, V. Trevino, H. Shen, P.W. Laird, D.A. Levine, S.L. Carter, G. Getz, K. Stemke-Hale, G.B. Mills, R.G. Verhaak, Inferring tumour purity and stromal and immune cell admixture from expression data, Nat Commun, 4 (2013) 2612.

[10] P. Charoentong, F. Finotello, M. Angelova, C. Mayer, M. Efremova, D. Rieder, H. Hackl, Z. Trajanoski, Pan-cancer Immunogenomic Analyses Reveal Genotype-Immunophenotype Relationships and Predictors of Response to Checkpoint Blockade, Cell Rep, 18 (2017) 248-262.

[11] A.M. Newman, C.L. Liu, M.R. Green, A.J. Gentles, W. Feng, Y. Xu, C.D. Hoang, M. Diehn, A.A. Alizadeh, Robust enumeration of cell subsets from tissue expression profiles, Nat Methods, 12 (2015) 453-457.

[12] D. Aran, Z. Hu, A.J. Butte, xCell: digitally portraying the tissue cellular heterogeneity landscape, Genome Biol, 18 (2017) 220.

[13] T. Li, J. Fan, B. Wang, N. Traugh, Q. Chen, J.S. Liu, B. Li, X.S. Liu, TIMER: A Web Server for Comprehensive Analysis of Tumor-Infiltrating Immune Cells, Cancer Res, 77 (2017) e108-e110.

[14] F. Finotello, C. Mayer, C. Plattner, G. Laschober, D. Rieder, H. Hackl, A. Krogsdam, Z. Loncova, W. Posch, D. Wilflingseder, S. Sopper, M. Ijsselsteijn, T.P. Brouwer, D. Johnson, Y. Xu, Y. Wang, M.E. Sanders, M.V. Estrada, P. Ericsson-Gonzalez, P. Charoentong, J. Balko, N. de Miranda, Z. Trajanoski, Molecular and pharmacological modulators of the tumor immune contexture revealed by deconvolution of RNA-seq data, Genome Med, 11 (2019) 34.

[15] E. Becht, N.A. Giraldo, L. Lacroix, B. Buttard, N. Elarouci, F. Petitprez, J. Selves, P. Laurent-Puig, C. Sautes-Fridman, W.H. Fridman, A. de Reynies, Estimating the population abundance of tissue-infiltrating immune and stromal cell populations using gene expression, Genome Biol, 17 (2016) 218.

[16] Y. Xiao, D. Ma, S. Zhao, C. Suo, J. Shi, M.Z. Xue, M. Ruan, H. Wang, J. Zhao, Q. Li, P. Wang, L. Shi, W.T. Yang, W. Huang, X. Hu, K.D. Yu, S. Huang, F. Bertucci, Y.Z. Jiang, Z.M. Shao, A.M.E.B.C.C. Group, Multi-Omics Profiling Reveals Distinct Microenvironment Characterization and Suggests Immune Escape Mechanisms of Triple-Negative Breast Cancer, Clin Cancer Res, 25 (2019) 5002-5014.

[17] P. Jiang, S. Gu, D. Pan, J. Fu, A. Sahu, X. Hu, Z. Li, N. Traugh, X. Bu, B. Li, J. Liu, G.J. Freeman, M.A. Brown, K.W. Wucherpfennig, X.S. Liu, Signatures of T cell dysfunction and exclusion predict cancer immunotherapy response, Nat Med, 24 (2018) 1550-1558.

[18] D. Zeng, Z. Ye, R. Shen, G. Yu, J. Wu, Y. Xiong, R. Zhou, W. Qiu, N. Huang, L. Sun, X. Li, J. Bin, Y. Liao, M. Shi, W. Liao, IOBR: Multi-Omics Immuno-Oncology Biological Research to Decode Tumor Microenvironment and Signatures, Front Immunol, 12 (2021) 687975.

[19] L. Xu, C. Deng, B. Pang, X. Zhang, W. Liu, G. Liao, H. Yuan, P. Cheng, F. Li, Z. Long, M. Yan, T. Zhao, Y. Xiao, X. Li, TIP: A Web Server for Resolving Tumor Immunophenotype Profiling, Cancer Res, 78 (2018) 6575-6580.

[20] R. Akbani, P.K. Ng, H.M. Werner, M. Shahmoradgoli, F. Zhang, Z. Ju, W. Liu, J.Y. Yang, K. Yoshihara, J. Li, S. Ling, E.G. Seviour, P.T. Ram, J.D. Minna, L. Diao, P. Tong, J.V. Heymach, S.M. Hill, F. Dondelinger, N. Stadler, L.A. Byers, F. Meric-Bernstam, J.N. Weinstein, B.M. Broom, R.G. Verhaak, H. Liang, S. Mukherjee, Y. Lu, G.B. Mills, A pan-cancer proteomic perspective on The Cancer Genome Atlas, Nat Commun, 5 (2014) 3887.

[21] Y. Ye, Y. Xiang, F.M. Ozguc, Y. Kim, C.J. Liu, P.K. Park, Q. Hu, L. Diao, Y. Lou, C. Lin, A.Y. Guo, B. Zhou, L. Wang, Z. Chen, J.S. Takahashi, G.B. Mills, S.H. Yoo, L. Han, The Genomic Landscape and Pharmacogenomic Interactions of Clock Genes in Cancer Chronotherapy, Cell Syst, 6 (2018) 314-328 e312.

[22] S. Hanzelmann, R. Castelo, J. Guinney, GSVA: gene set variation analysis for microarray and RNA-seq data, BMC Bioinformatics, 14 (2013) 7.

[23] L. Wang, Z. Liu, R. Liang, W. Wang, R. Zhu, J. Li, Z. Xing, S. Weng, X. Han, Y.L. Sun, Comprehensive machine-learning survival framework develops a consensus model in large-scale multicenter cohorts for pancreatic cancer, Elife, 11 (2022).

[24] Z. Liu, L. Liu, S. Weng, C. Guo, Q. Dang, H. Xu, L. Wang, T. Lu, Y. Zhang, Z. Sun, X. Han, Machine learning-based integration develops an immune-derived lncRNA signature for improving outcomes in colorectal cancer, Nat Commun, 13 (2022) 816.

[25] Z. Liu, C. Guo, Q. Dang, L. Wang, L. Liu, S. Weng, H. Xu, T. Lu, Z. Sun, X. Han, Integrative analysis from multi-center studies identities a consensus machine learning-derived lncRNA signature for stage II/III colorectal cancer, EBioMedicine, 75 (2022) 103750.

[26] S. Mariathasan, S.J. Turley, D. Nickles, A. Castiglioni, K. Yuen, Y. Wang, E.E. Kadel, III, H. Koeppen, J.L. Astarita, R. Cubas, S. Jhunjhunwala, R. Banchereau, Y. Yang, Y. Guan, C. Chalouni, J. Ziai, Y. Senbabaoglu, S. Santoro, D. Sheinson, J. Hung, J.M. Giltnane, A.A. Pierce, K. Mesh, S. Lianoglou, J. Riegler, R.A.D. Carano, P. Eriksson, M. Hoglund, L. Somarriba, D.L. Halligan, M.S. van der Heijden, Y. Loriot, J.E. Rosenberg, L. Fong, I. Mellman, D.S. Chen, M. Green, C. Derleth, G.D. Fine, P.S. Hegde, R. Bourgon, T. Powles, TGFbeta attenuates tumour response to PD-L1 blockade by contributing to exclusion of T cells, Nature, 554 (2018) 544-548.

[27] N. Riaz, J.J. Havel, V. Makarov, A. Desrichard, W.J. Urba, J.S. Sims, F.S. Hodi, S. Martin-Algarra, R. Mandal, W.H. Sharfman, S. Bhatia, W.J. Hwu, T.F. Gajewski, C.L. Slingluff, Jr., D. Chowell, S.M. Kendall, H. Chang, R. Shah, F. Kuo, L.G.T. Morris, J.W. Sidhom, J.P. Schneck, C.E. Horak, N. Weinhold, T.A. Chan, Tumor and Microenvironment Evolution during Immunotherapy with Nivolumab, Cell, 171 (2017) 934-949 e916.

[28] E.M. Van Allen, D. Miao, B. Schilling, S.A. Shukla, C. Blank, L. Zimmer, A. Sucker, U. Hillen, M.H.G. Foppen, S.M. Goldinger, J. Utikal, J.C. Hassel, B. Weide, K.C. Kaehler, C. Loquai, P. Mohr, R. Gutzmer, R. Dummer, S. Gabriel, C.J. Wu, D. Schadendorf, L.A. Garraway, Genomic correlates of response to CTLA-4 blockade in metastatic melanoma, Science, 350 (2015) 207-211.

[29] D.A. Braun, Y. Hou, Z. Bakouny, M. Ficial, M. Sant' Angelo, J. Forman, P. Ross-Macdonald, A.C. Berger, O.A. Jegede, L. Elagina, J. Steinharter, M. Sun, M. Wind-Rotolo, J.C. Pignon, A.D. Cherniack, L. Lichtenstein, D. Neuberg, P. Catalano, G.J. Freeman, A.H. Sharpe, D.F. McDermott, E.M. Van Allen, S. Signoretti, C.J. Wu, S.A. Shukla, T.K. Choueiri, Interplay of somatic alterations and immune infiltration modulates response to PD-1 blockade in advanced clear cell renal cell carcinoma, Nat Med, 26 (2020) 909-918.

[30] T.N. Gide, C. Quek, A.M. Menzies, A.T. Tasker, P. Shang, J. Holst, J. Madore, S.Y. Lim, R. Velickovic, M. Wongchenko, Y. Yan, S. Lo, M.S. Carlino, A. Guminski, R.P.M. Saw, A. Pang, H.M. McGuire, U. Palendira, J.F. Thompson, H. Rizos, I.P.D. Silva, M. Batten, R.A. Scolyer, G.V. Long, J.S. Wilmott, Distinct Immune Cell Populations Define Response to Anti-PD-1 Monotherapy and Anti-PD-1/Anti-CTLA-4 Combined Therapy, Cancer Cell, 35 (2019) 238-255 e236.

[31] J. Zhao, A.X. Chen, R.D. Gartrell, A.M. Silverman, L. Aparicio, T. Chu, D. Bordbar, D. Shan, J. Samanamud, A. Mahajan, I. Filip, R. Orenbuch, M. Goetz, J.T. Yamaguchi, M. Cloney, C. Horbinski, R.V. Lukas, J. Raizer, A.I. Rae, J. Yuan, P. Canoll, J.N. Bruce, Y.M. Saenger, P. Sims, F.M. Iwamoto, A.M. Sonabend, R. Rabadan, Author Correction: Immune and genomic correlates of response to anti-PD-1 immunotherapy in glioblastoma, Nat Med, 25 (2019) 1022.

[32] Z. Chen, Z. Luo, D. Zhang, H. Li, X. Liu, K. Zhu, H. Zhang, Z. Wang, P. Zhou, J. Ren, A. Zhao, Z. Zuo, TIGER: A Web Portal of Tumor Immunotherapy Gene Expression Resource, Genomics Proteomics Bioinformatics, (2022).

[33] Y. Hoshida, J.P. Brunet, P. Tamayo, T.R. Golub, J.P. Mesirov, Subclass mapping: identifying common subtypes in independent disease data sets, PLoS One, 2 (2007) e1195.

[34] X. Lu, L. Jiang, L. Zhang, Y. Zhu, W. Hu, J. Wang, X. Ruan, Z. Xu, X. Meng, J. Gao, X. Su, F. Yan, Immune Signature-Based Subtypes of Cervical Squamous Cell Carcinoma Tightly Associated with Human Papillomavirus Type 16 Expression, Molecular Features, and Clinical Outcome, Neoplasia, 21 (2019) 591-601.

[35] Z. Zeng, X. Zhang, D. Li, J. Li, J. Yuan, L. Gu, X. Xiong, Expression, Location, Clinical Implication, and Bioinformatics Analysis of RNASET2 in Gastric Adenocarcinoma, Front Oncol, 10 (2020) 836.

[36] Z. Zeng, X. Zhang, C.Q. Jiang, Y.G. Zhang, X. Wu, J. Li, S. Tang, L. Li, L.J. Gu, X.Y. Xie, Y.A. Jiang, Identifying novel therapeutic targets in gastric cancer using genome-wide CRISPR-Cas9 screening, Oncogene, 41 (2022) 2069-2078.

**Supplementary Figures**


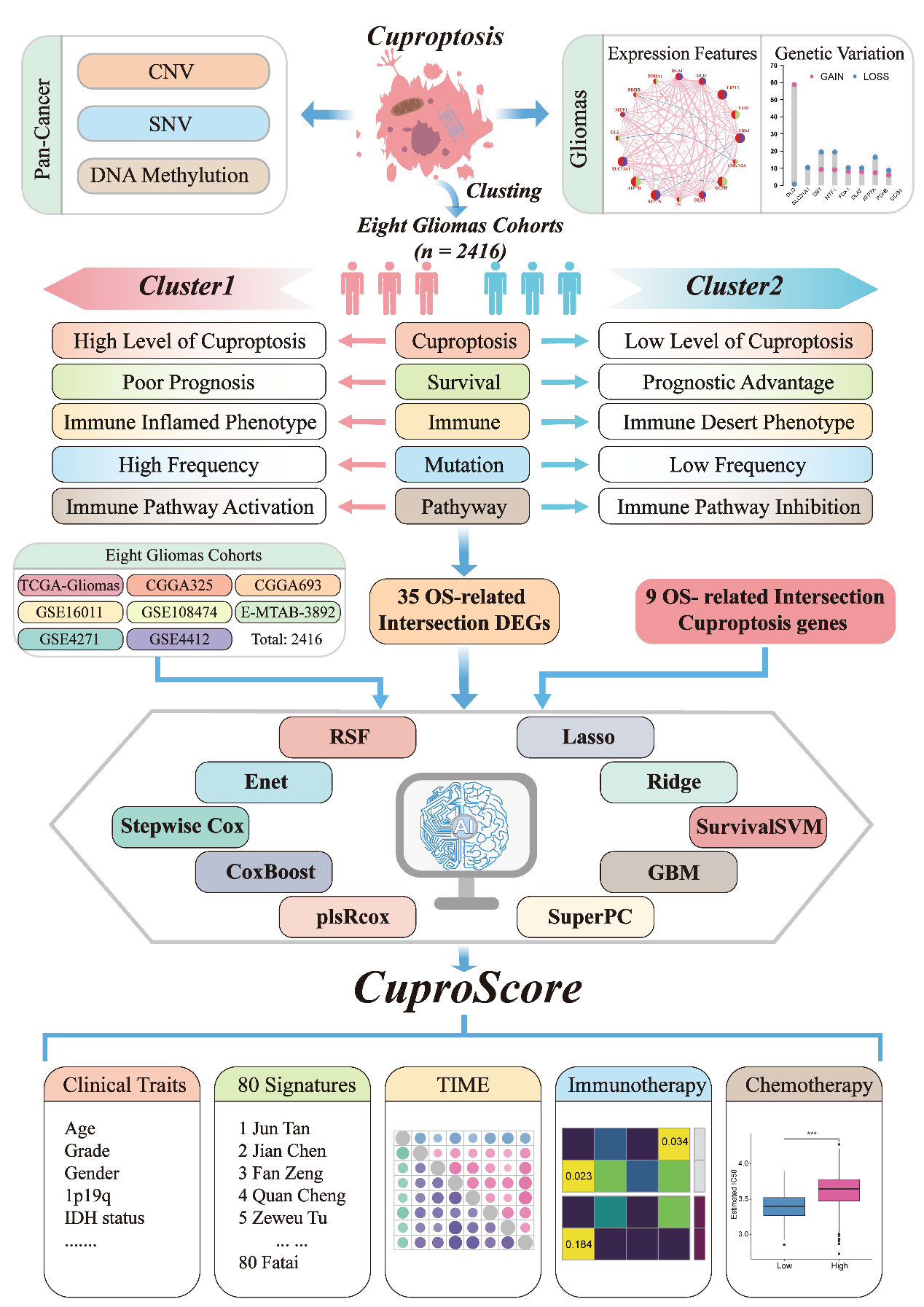


**Figure S1. The workflow of our research.**


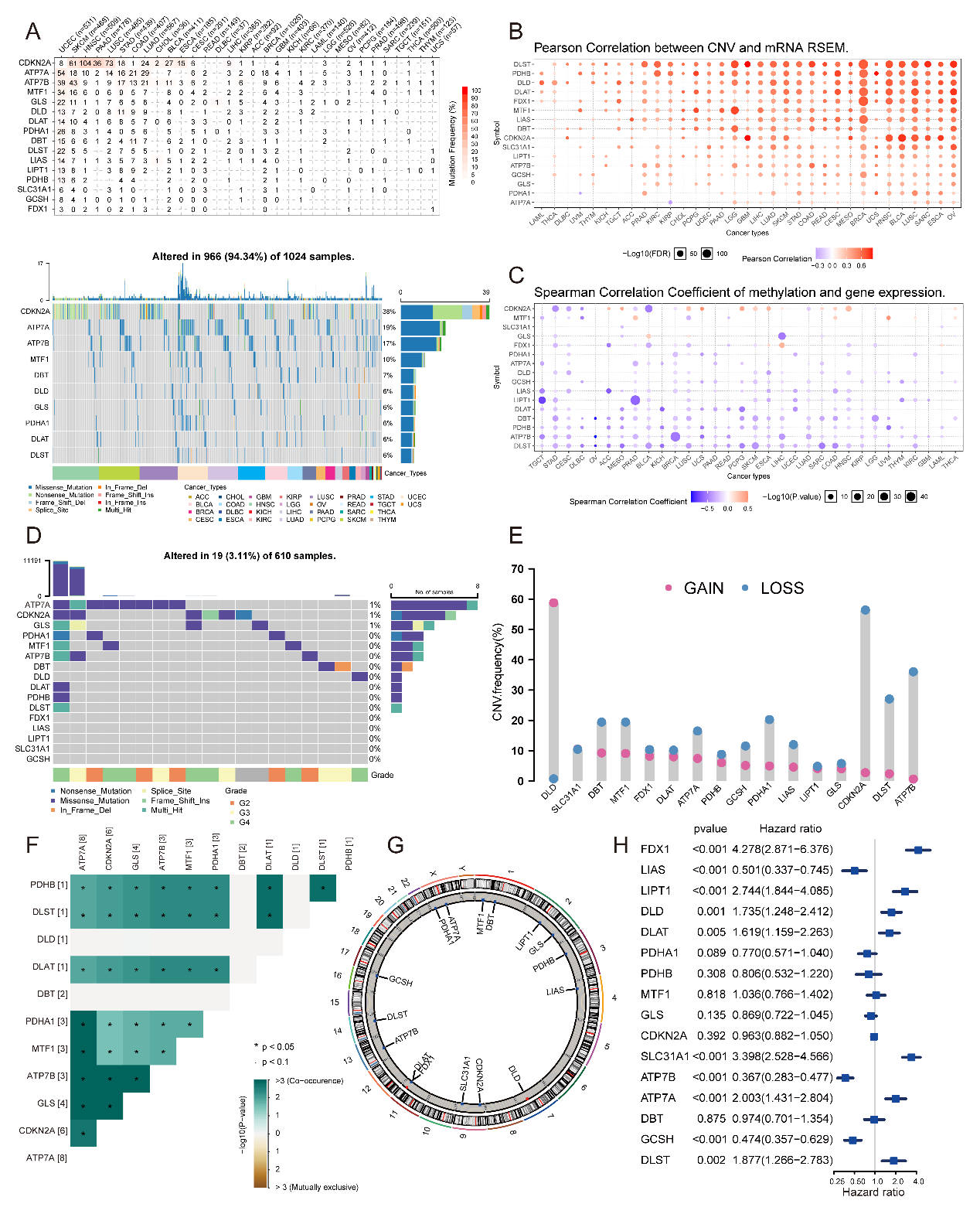


**Figure S2. The epigenetic landscape of cuproptosis molecule alterations in glioma.**

(A) The mutation frequency heatmap (upper part) displayed the mutation rate of cuproptosis molecules in pan-cancer. Blanks indicate that all regions of the gene are free of mutation. The SNV oncoplot (bottom part) represented the SNV frequency, types, and distribution of cuproptosis molecules in pan-cancer. (B-C) The correlation heatmap showed the correlation of mRNA levels of cuproptosis molecules with copy number variation (B) and DNA methylation (C), respectively. Red represents a positive correlation, whereas blue represents a negative correlation. The deeper the color, the greater the correlation index. The size of the bubble determines the FDR. (D) The waterfall plot showed the SNV frequency, types, and distribution of the cuproptosis molecules in 610 glioma patients. (E) Copy number variation frequency among glioma patients in the TCGA cohort. Red and blue reflect copy numbers gained and lost, respectively. (F) The correlation heatmap exhibited mutational associations of cuproptosis molecules. Green and brown colors represented co-mutations and mutex-mutation, respectively. The asterisk indicated P-value (*p < 0.05, ·p < 0.1). (G) The location of cuproptosis genes with CNV alteration on chromosomes. (H) The forest plot displayed the results of a univariate Cox regression analysis on the average survival of 16 cuproptosis molecules in the TCGA cohort.


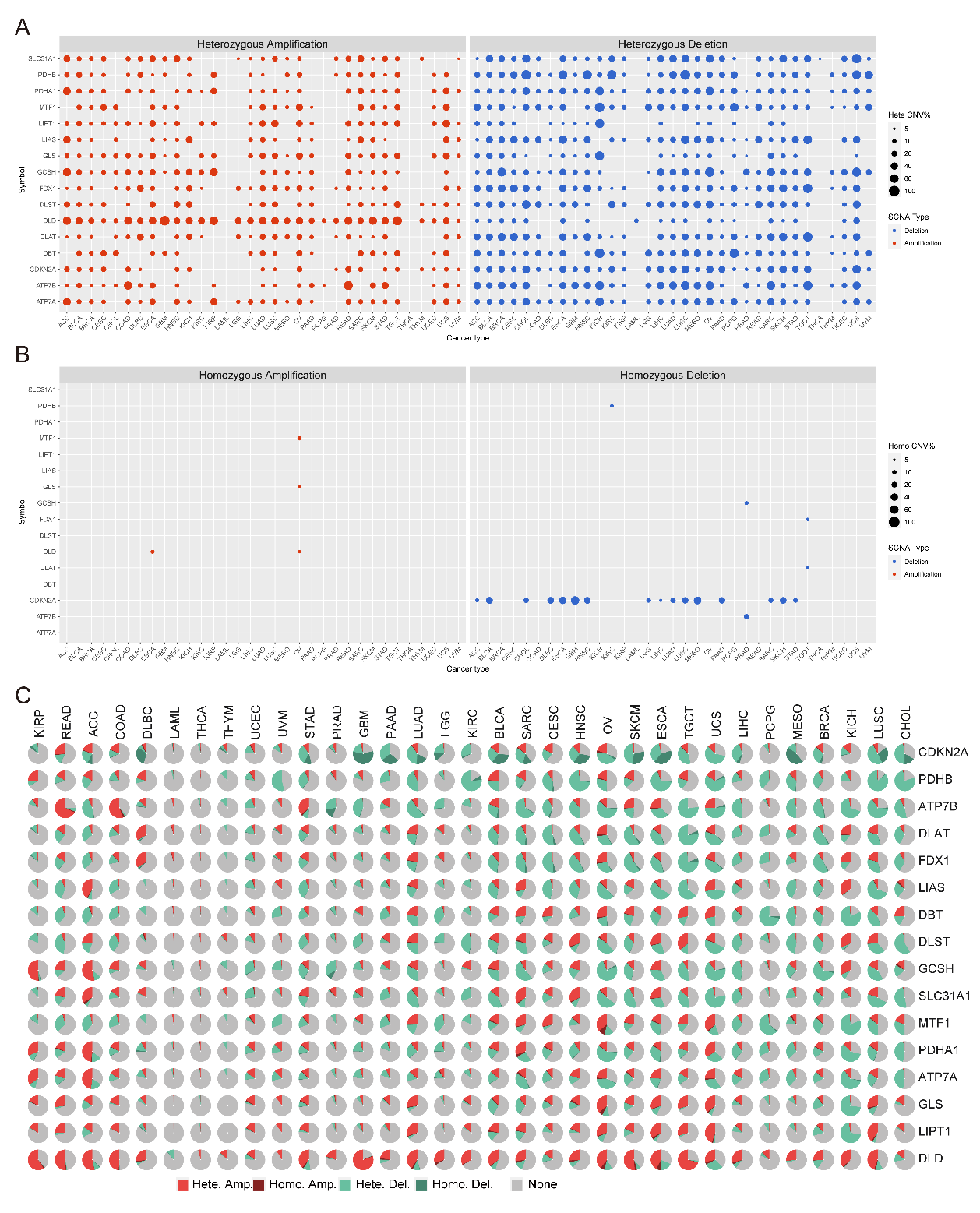


**Figure S3. Pan-cancer analysis of genetic changes in cuproptosis-related molecules**

(A-B) The CNV bubble chart depicts the distribution of (A) heterozygous and (B) homozygous CNV of cuproptosis molecules in each cancer. (C) The CNV pie distribution illustrates the heterozygous/homozygous CNV distribution of each gene in each tumor. A pie represents the proportion of CNV types of cuproptosis molecules in a cancer type; different colors represent different CNV types. Hete Amp: heterozygous amplification; Hete Del: heterozygous deletion; Homo Amp: homozygous amplification; Homo Del: homozygous deletion; None: no CNV.


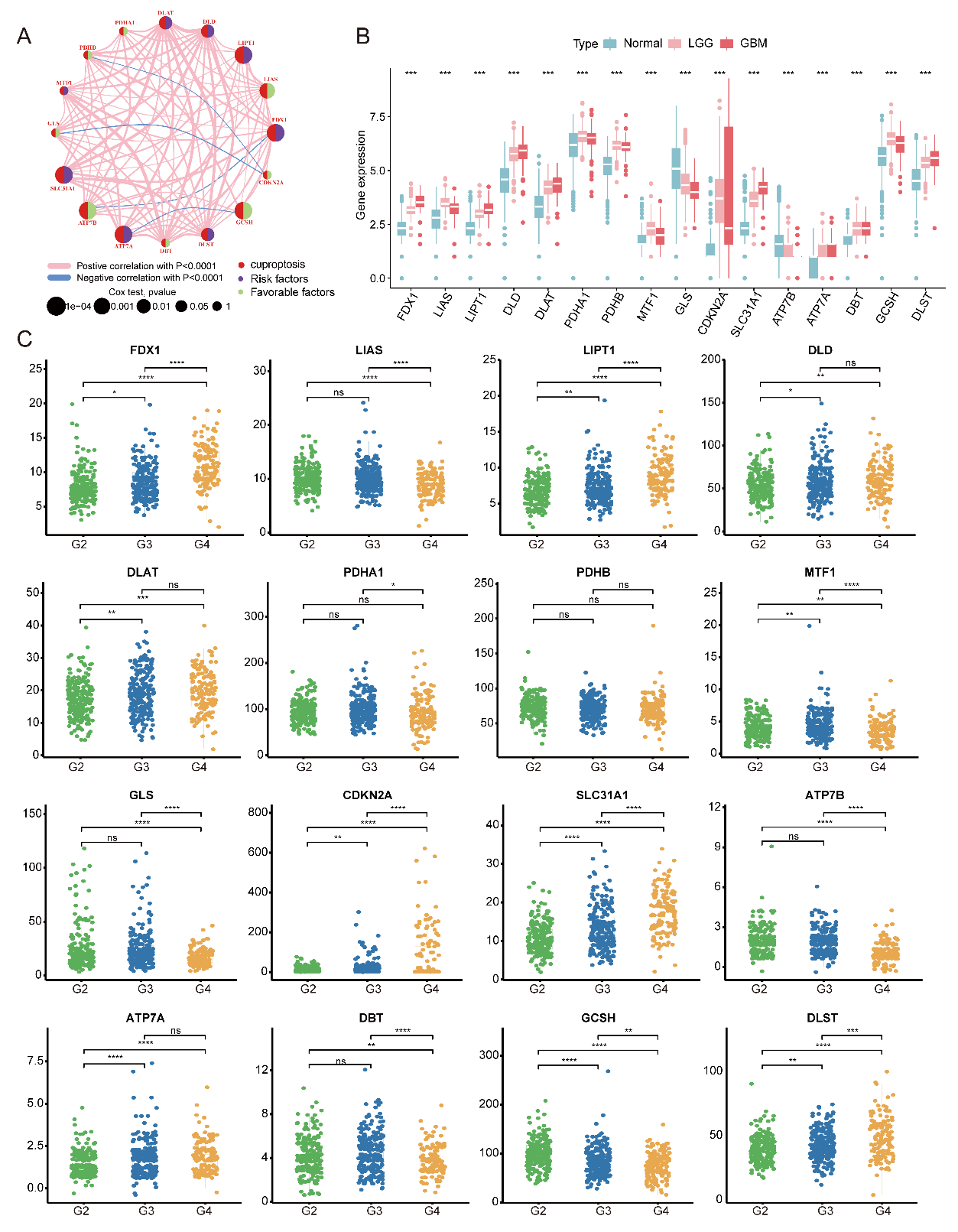


**Figure S4. Cuproptosis regulatory molecules and clinical features of glioma patients.**

**(**A) The network showed interactions among Cuproptosis molecules in gliomas in the TCGA cohort. (**B**) The mRNA expression level of cuproptosis genes among normal, TCGA-LGG, and TCGA-GBM samples. The asterisks represented the statistical p-value (Kruskal–Wallis test: **p* < 0.05, ** *p* < 0.001, *** *p* < 0.001). (C) Significant differentially expressed cuproptosis genes among distinct pathologic stages were examined. The asterisks represented the statistical p-value (Kruskal–Wallis test: *p <

0.05, ** p < 0.01, *** p < 0.005, **** p < 0.001).


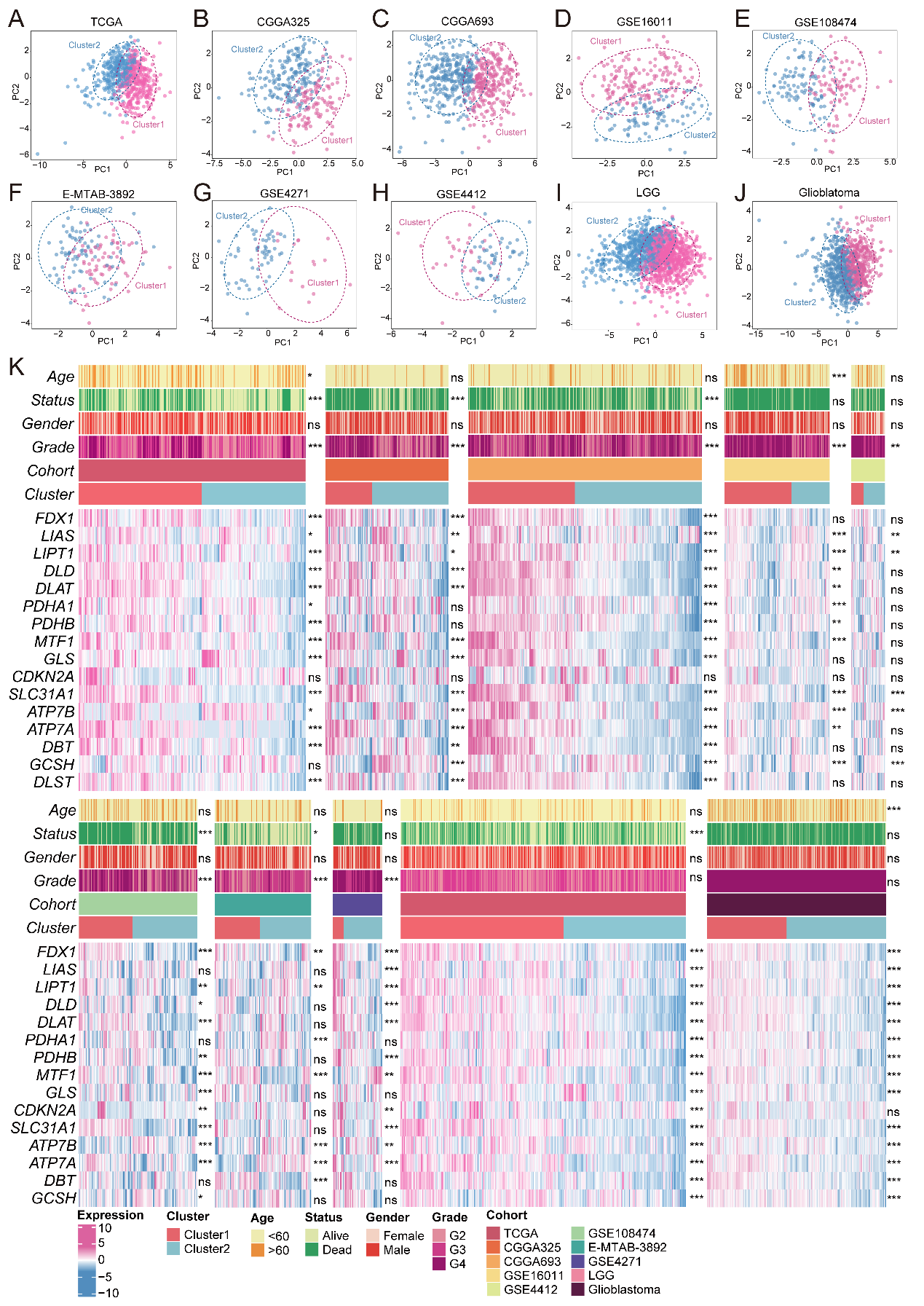


**Figure S5. Track of the unsupervised K-means-based consensus-clustering**

(A-J) PCA for the transcriptome profiles of cuproptosis patterns in the TCGA-Gliomas (A), CGGA325 (B), CGGA693 (C), GSE16011 (D), GSE108474 (E), E-MTAB-3892 (F), GSE4271 (G), GSE4412 (H), LGG (I), and glioblastoma (J) cohorts. (K) This heatmap demonstrates the relationships between the two cuproptosis phenotypes, clinicopathologic characteristics, and the expression variations of the cuproptosis-related genes in ten cohorts. The top portion represented Fisher's precise test. The lower portion indicated the Wilcoxon rank-sum test. ****p* < 0.001, ***p* < 0.01, **p* < 0.05, and "ns" stood for no statistical significance.


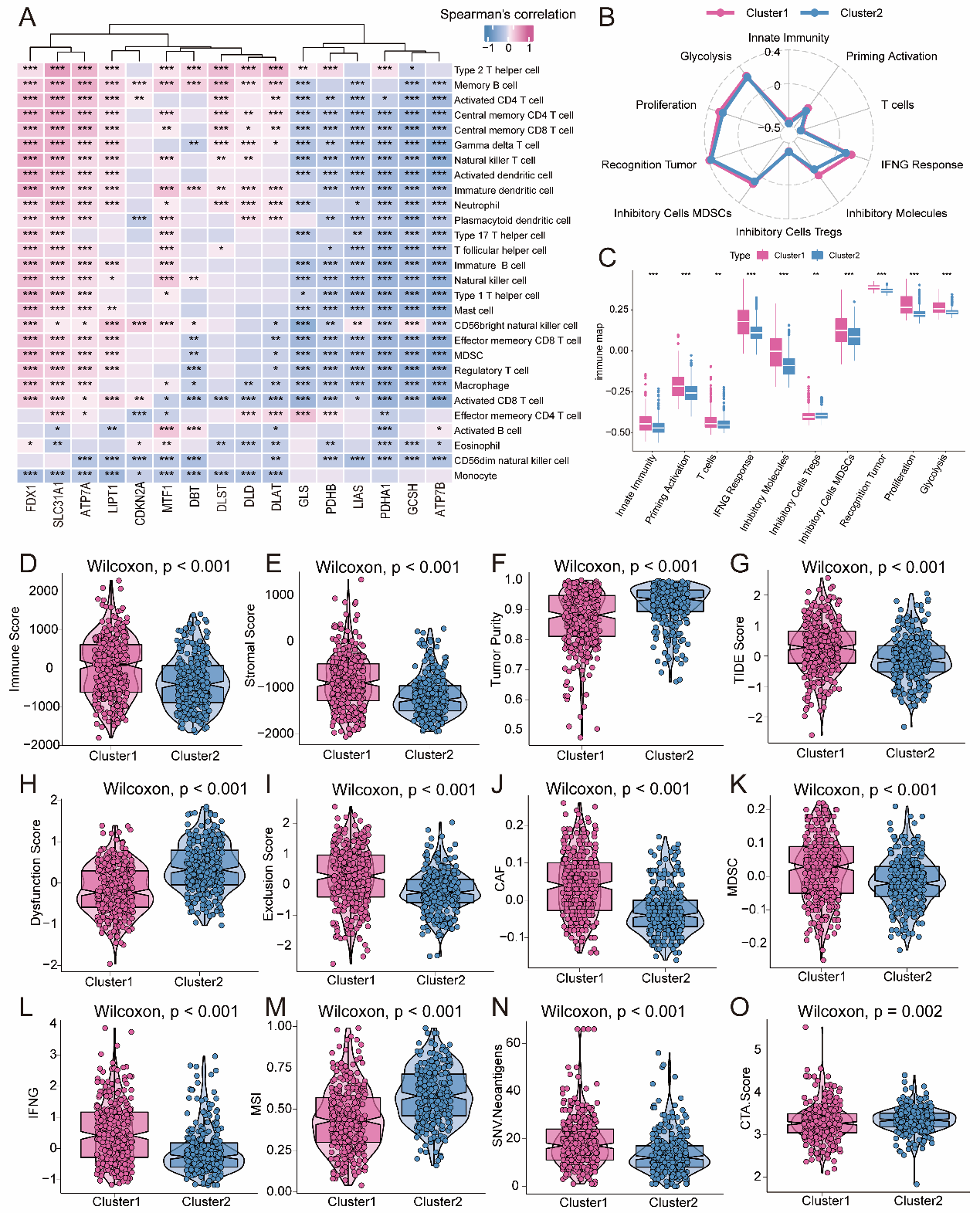


**Figure S6. Track of the quantified tumor immune microenvironment.**

(A) The heatmap exhibited the correlation between mRNA expression levels of cuproptosis molecules and diverse types of immune infiltrating cells. The pink and blue colors represented positive and negative correlations, respectively. Asterisks denoted p-value (**p* < 0.05; ** *p* < 0.01, *** *p* < 0.001). Blank cells represented no statistical significance of the correlation. (B) Immunogram radar plot displaying the variances of TIME signatures developed by Kobayashi. (C) The Wilcoxon rank-sum test revealed the variances of TIME signatures developed by Kobayashi between two cuproptosis clusters (*p < 0.05; ** p < 0.001, *** p < 0.001). (D-O) Boxplots showed the differences in immune score (D), stromal score (E), tumor purity (F), TIDE (G), dysfunction score (H), exclusion score (I), CAF (J), MDSC (K), IFNG (L), MSI (M), SNV. neoantigens (N), and CTA. score (O) between the cuproptosis clusters. The points represented the raw data distribution. The line in the box represented the median value.


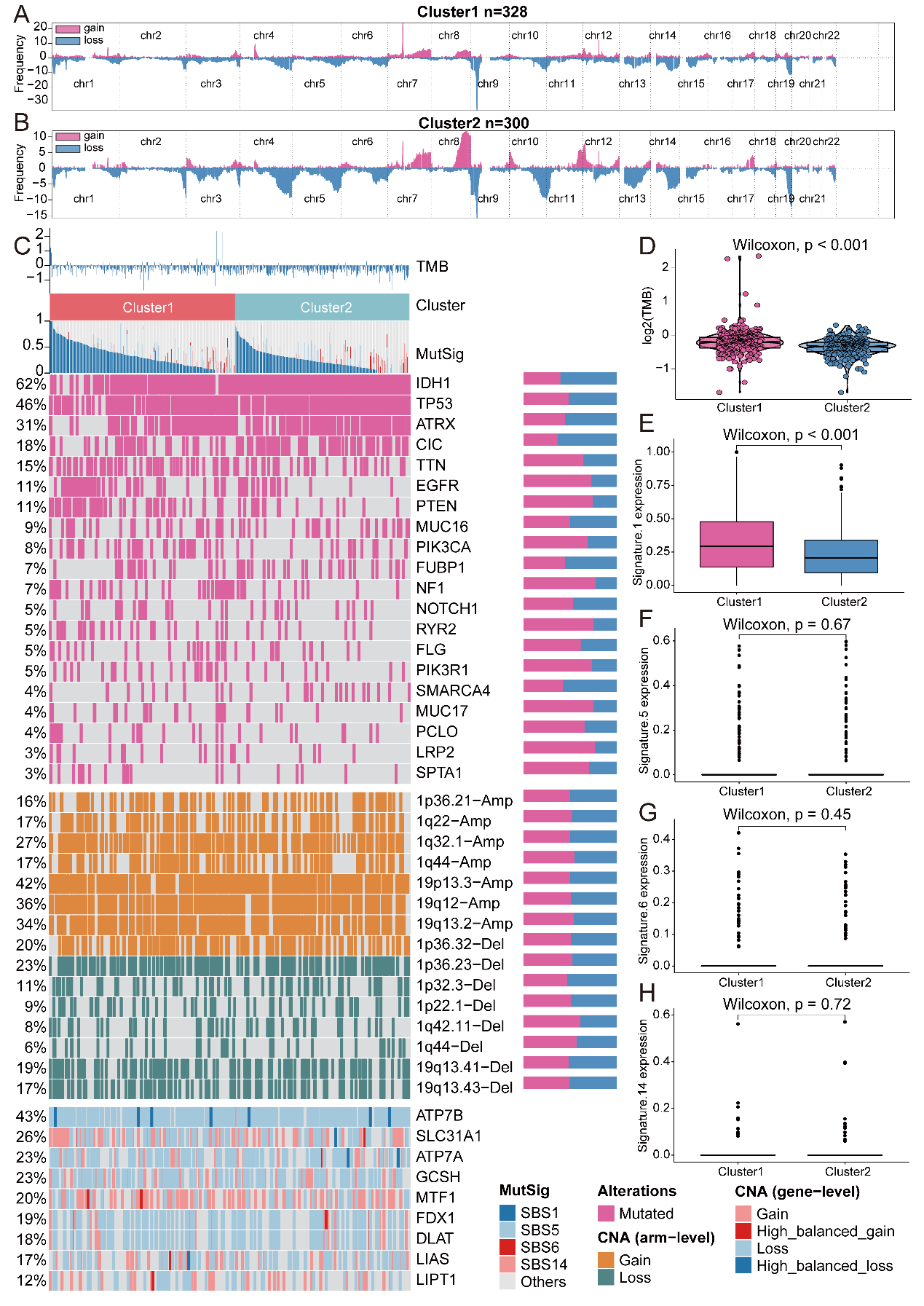


**Figure S7. The distinction of the genomic characteristics between the two cuproptosis phenotypes.**

(A-B) The plot illustrates the frequency of the gain (pink) and loss (blue) of each gene in Cluster1 (A) and Cluster2 (B). Gene segments are placed according to their location in chromosomes, ranging from chromosomes 1 to 22. (C) The landscape of somatic mutations based on cuproptosis clustering subtypes. TMB, the relative contribution of the four mutational signatures (SBS1, SBS5, SBS6, and SBS14), selected top-mutated genes, and top-broad-level copy number alterations (q-value 0.05), and selected cuproptosis molecules were displayed in ascending order from the top panel to the bottom. In the right stacked bar plots, the percentage of variation conversions was displayed. (D) TMB differences between the two clusters were revealed by Wilcoxon rank-sum test. (E-H) The four mutational signature variations between the two cuproptosis phenotypes were revealed by the Wilcoxon rank-sum test.


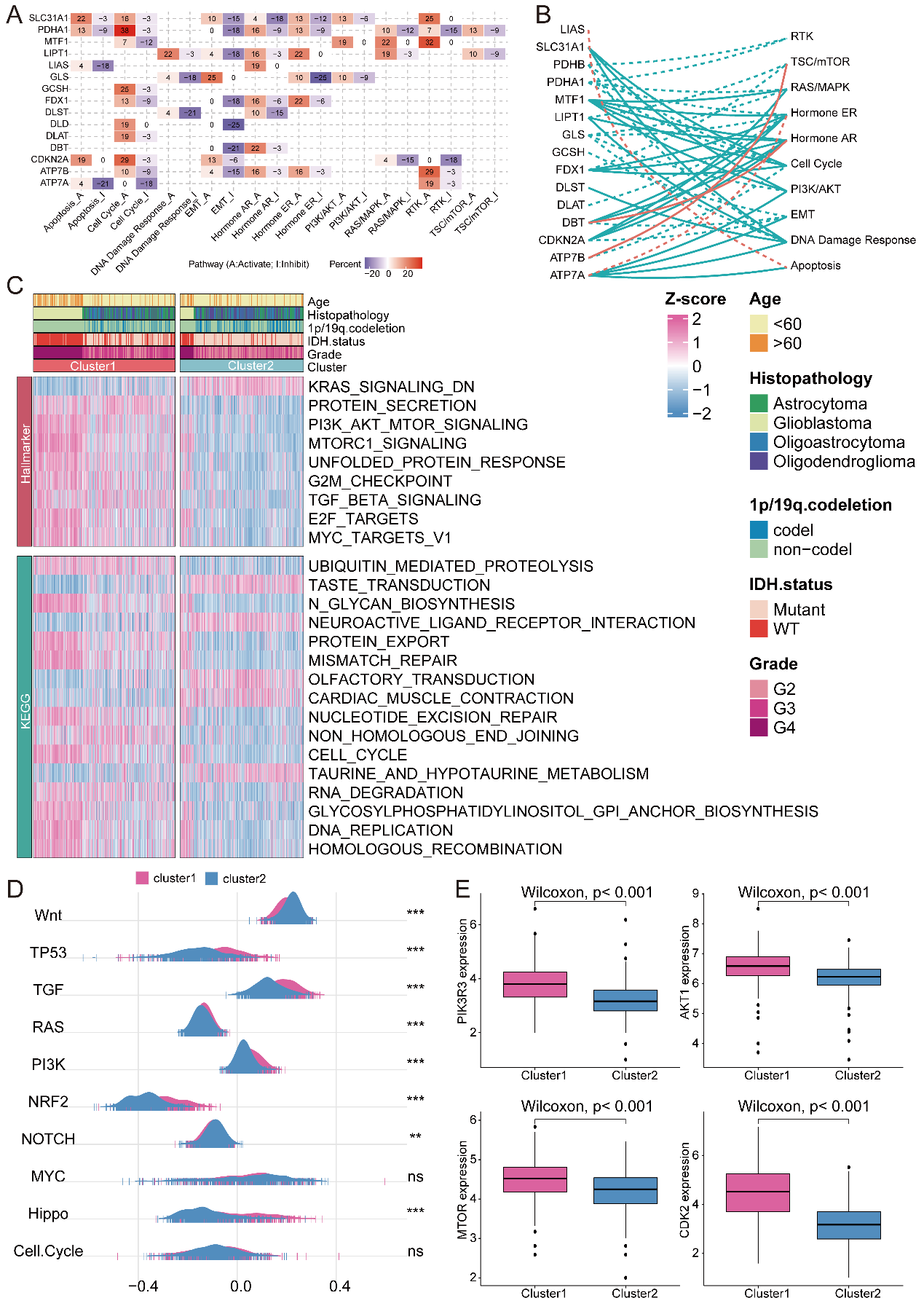


**Figure S8. Investigation of cuproptosis-related signal pathways.**

(A) This heatmap demonstrated the regulation of cuproptosis molecules on broadly acknowledged cancer-related pathways at the protein level. Only a function (inhibited or activated) was demonstrated in at least five cancer types. "Pathway activate" in red denoted the percentage of cancers where a pathway may be triggered by cuproptosis regulators, and "Pathway inhibit" in blue represented inhibition in a similar manner. (B) The line connections illustrated the regulatory relationship between cuproptosis molecules and classical cancer pathways in the network. The solid line indicated activation, whereas the dotted line indicated inhibition. The green line indicated LGG, while the orange-red line indicated glioblastoma. (C) GSVA enrichment analyses in two cuproptosis clusters illustrated the activation status of Hallmark pathways and KEGG in TCGA cohorts. Pink and blue represent activation and inhibition of the pathway, respectively. (D) The ten vital broadly acknowledged cancer-related signaling pathways between the two cuproptosis subtypes. Asterisks denoted p-value (***p < 0.001, Wilcoxon rank-sum test) (E) The levels of PI3K/AKT/mTOR signaling pathway between the two cuproptosis phenotypes examined by the Wilcoxon rank-sum test.


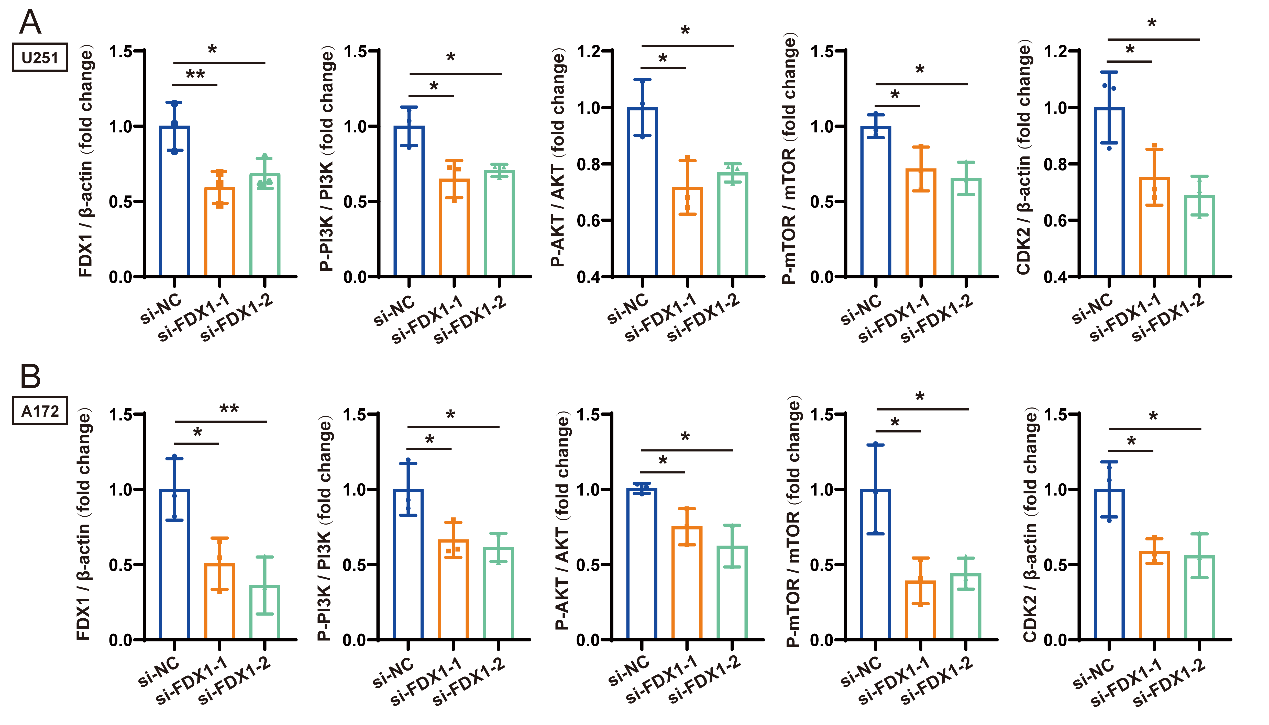


**Figure S9. Quantification results of the western blots of PI3K/AKT/mTOR signaling pathway after downregulation of FDX1.**

(A-B) Quantification results of FDX1, P-PI3K, PI3K, P-AKT, AKT, P-mTOR, mTOR, and CDK2 protein levels after the downregulation of FDX1 in U251 (A) and A172 (B). The asterisks represented the statistical p-value (one-way ANOVA test, * p < 0.05; ** p < 0.01, *** p < 0.001).


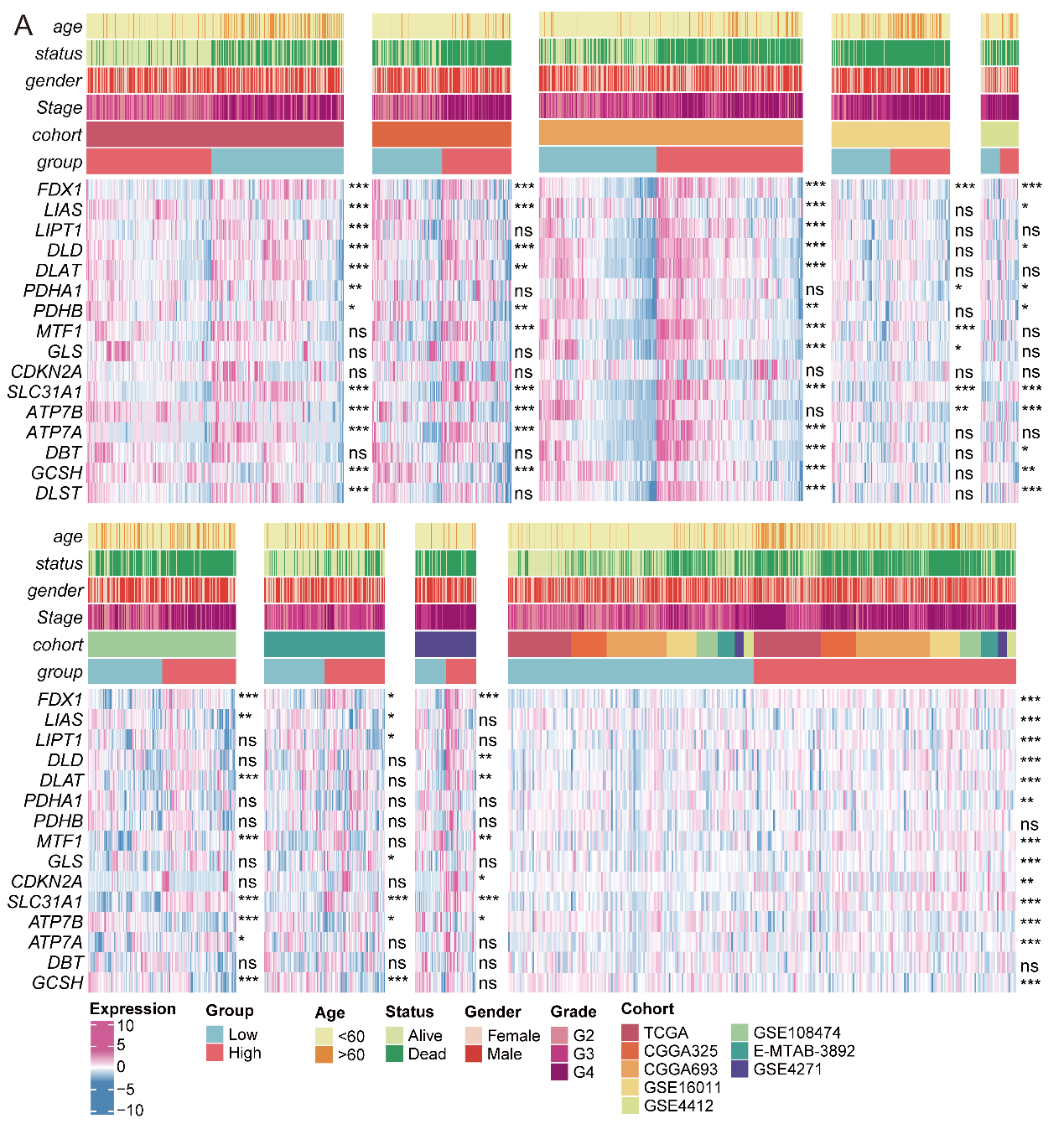


**Figure S10. The expression distribution of the cuproptosis-related genes between CuproScore groups.**

(A) This heatmap demonstrates the relationships between the two CuproScore groups and the expression variations of the cuproptosis-related genes in TCGA, CGGA325, CGGA693, GSE16011, GSE108474, E-MTAB-3892, GSE4271, GSE4412, and meta-cohort. The asterisks represented the statistical p-value (Wilcoxon rank-sum test, ****p* < 0.001, ***p* < 0.01, **p* < 0.05, and "ns" stood for no statistical significance.).


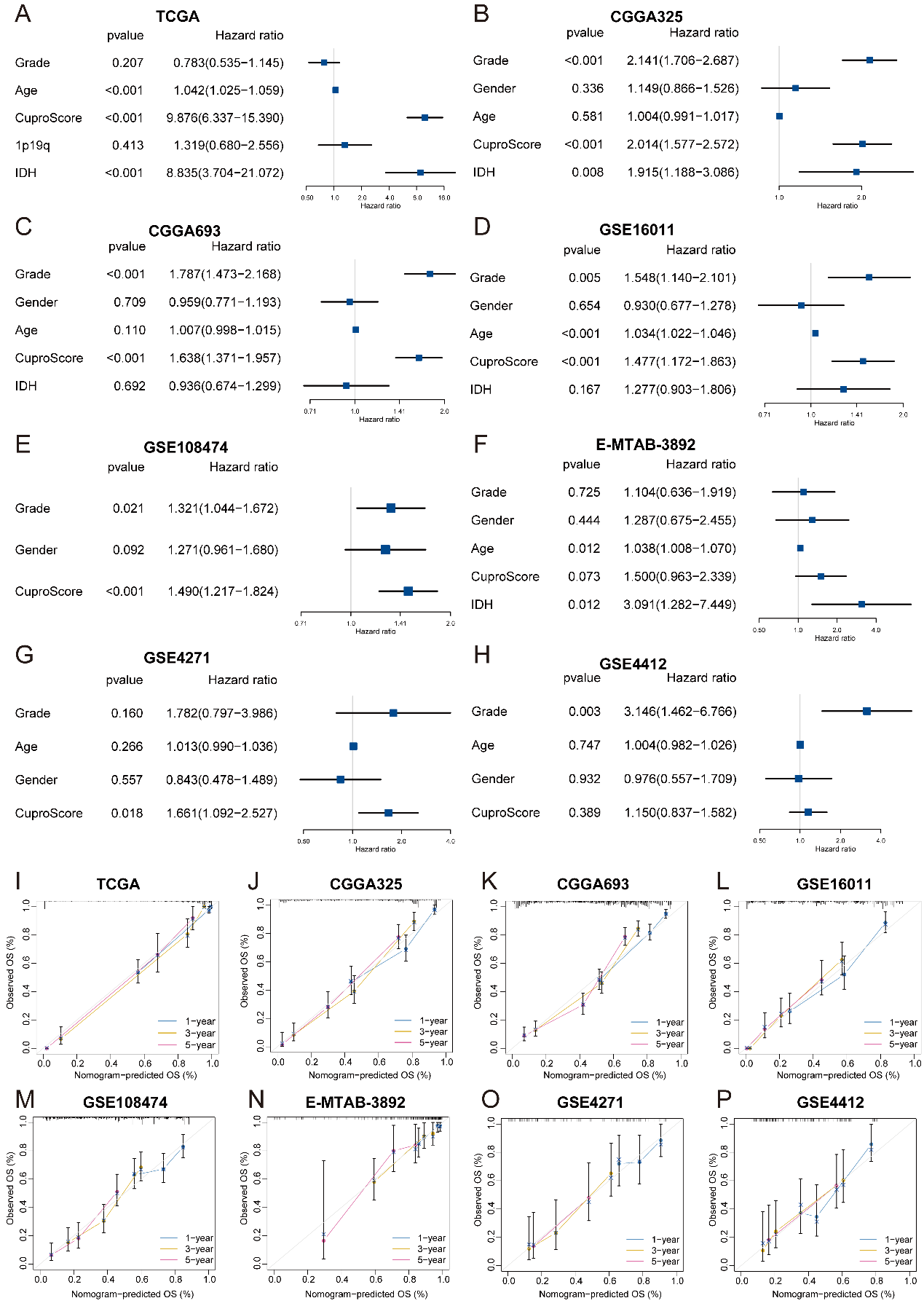


**Figure S11. Survival analysis of CuproScore in eight cohorts.**

(A-H) Multivariate Cox regression analysis of overall survival (OS) in the TCGA-Gliomas (A), CGGA325 (B), CGGA693 (C), GSE16011 (D), GSE108474 (E), E-MTAB-3892 (F), GSE4271 (G), and GSE4412(H). (I-P) Plots depicted the calibration of nomograms in the TCGA-Gliomas (I), CGGA325 (J), CGGA693 (K), GSE16011 (L), GSE108474 (M), E-MTAB-3892 (N), GSE4271 (O), and GSE4412(P).


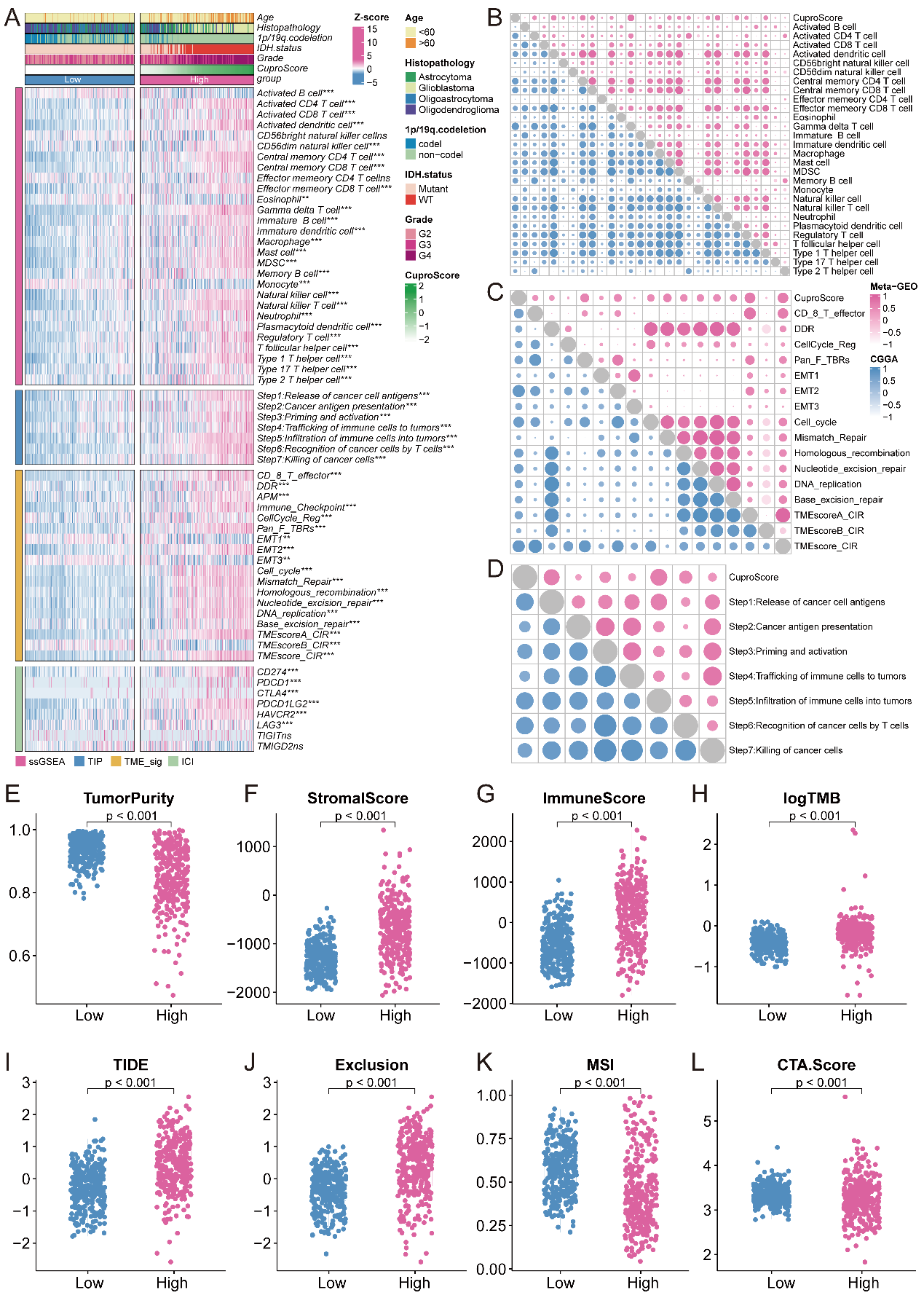


**Figure S12. Alterations in tumor immune microenvironments between high and low Cuproscore groups.**

(A) This complex heatmap displays the tumor immune microenvironment profile in the TCGA-Gliomas cohort, including immune infiltrating cell types, immune-related pathways, the enrichment levels of seven anti-tumor immune cycle steps, and immune checkpoints. Annotated clinicopathologic characteristics are at the top of the heatmap. (B-D) The correlations between Cuproscore and the ssGSEA scores of TIME cells (B), TIME-related pathways (C), and immune cycle steps (D) in glioma patients of Meta-GEO and CGGA cohort, respectively (*p* < 0.05). Pink represented positive correlation in Meta-GEO cohort; blue represented positive correlation in CGGA cohort. (E-L) Box plots showed the significant difference in tumor purity (E), stromal score (F), immune score (G), TMB (H), TIDE (I), exclusion score (J), MSI (K) and CTA. score (L) between high and low Cuproscore groups.


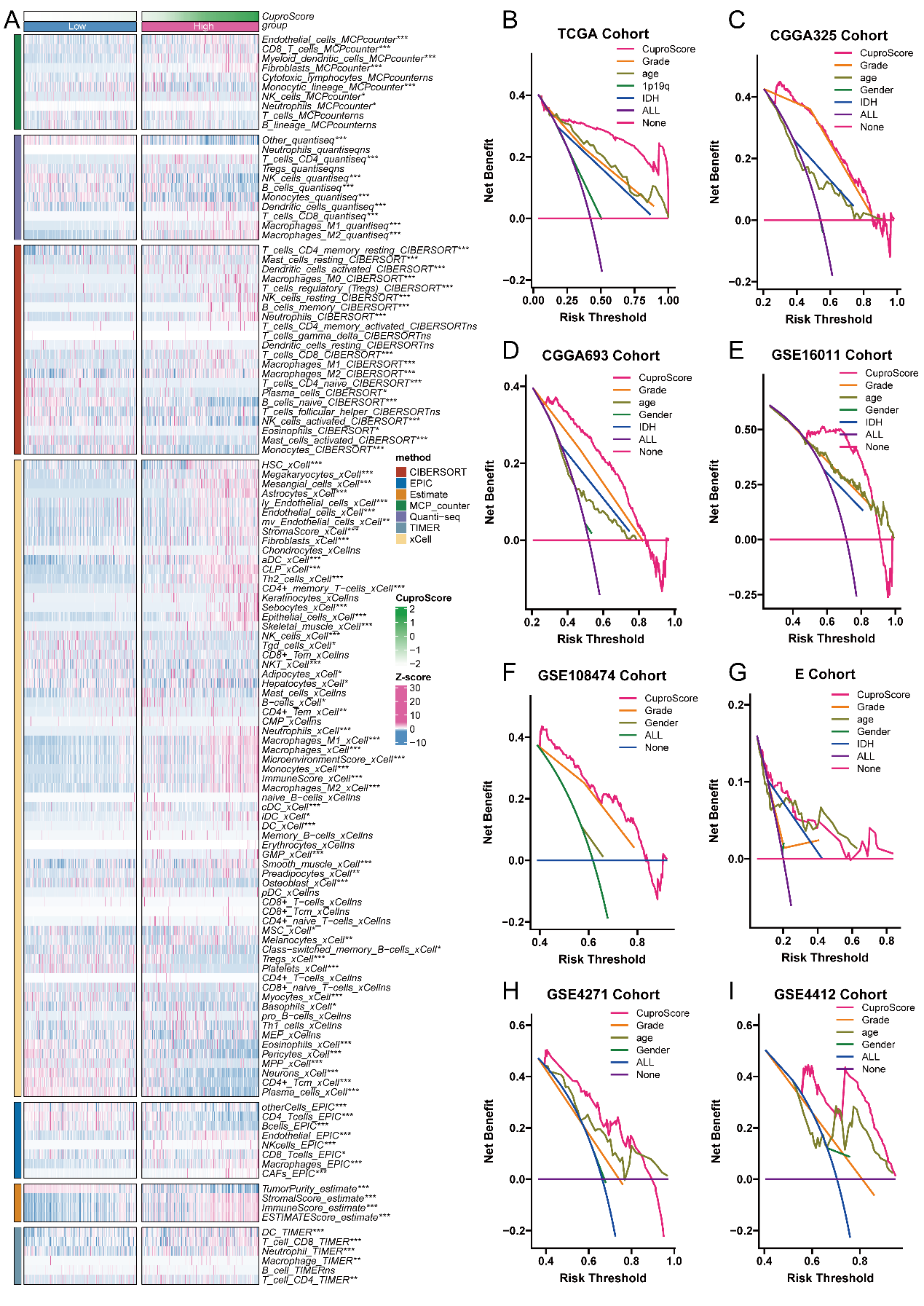


**Figure S13. The correlation between immune characteristics and the CuproScore.**

(A) The heatmap showed the associations between the CuproScore and immunocyte infiltrating levels by different methods. (B-I) Decision analysis showed the clinical efficiency of the CuproScore in TCGA-Gliomas (B), CGGA325 (C), CGGA693 (D), GSE16011 (E), GSE108474 (F), E-MTAB-3892 (G), GSE4271 (H), and GSE4412 (I) at 1, 3, and 5 years. The CuproScore exhibited the highest net benefit stably.


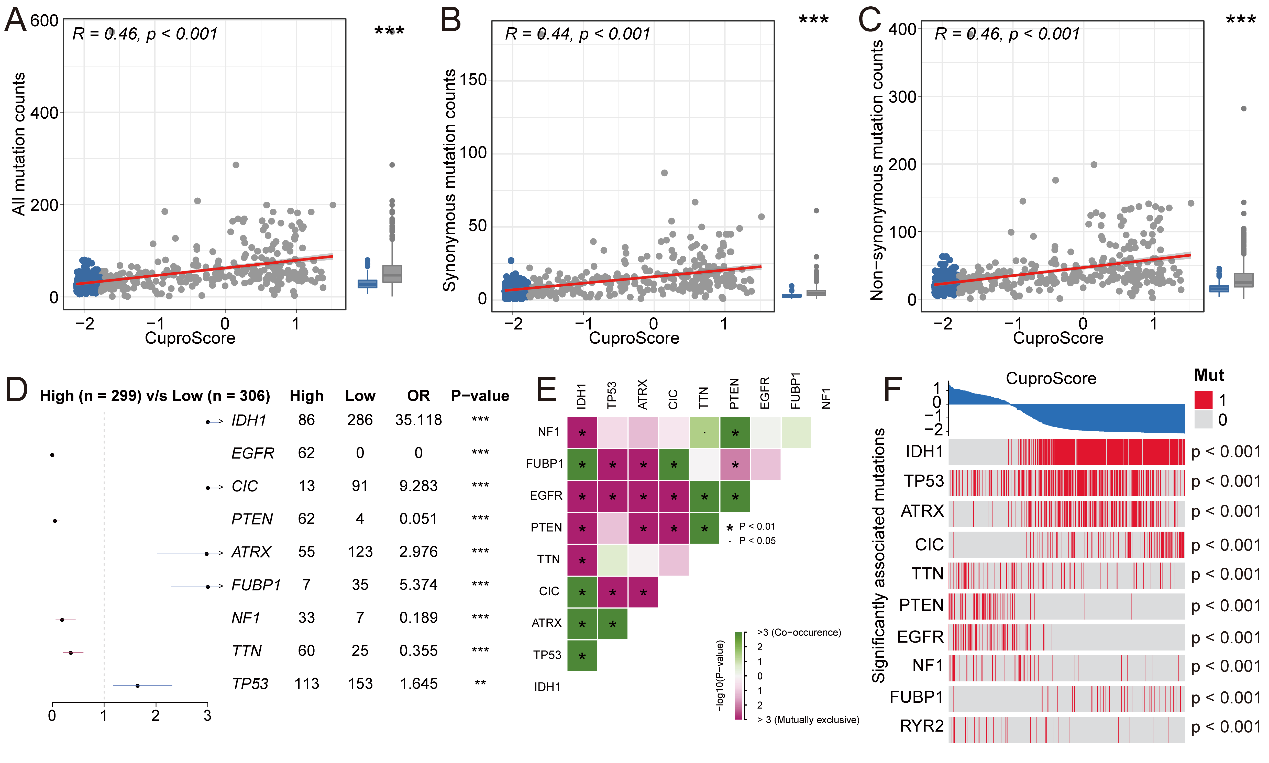


**Figure S14. The CuproScore was correlated to tumor mutation status.**

(A-C) The relationship between all mutation, synonymous, non-synonymous counts and the CuproScore in the low and high CuproScore groups. Blue represented low CuproScore patients, and grey represented high CuproScore patients. (D) A forest plot showed differentially mutated genes in glioma patients of the low and high CuproScore groups. (E) Interplay effects of differentially mutated genes in glioma patients including co-occurrence and mutual exclusion. (F) The correlation between the CuproScore and mutations of selected top-mutated genes in glioma revealed by permutation test.


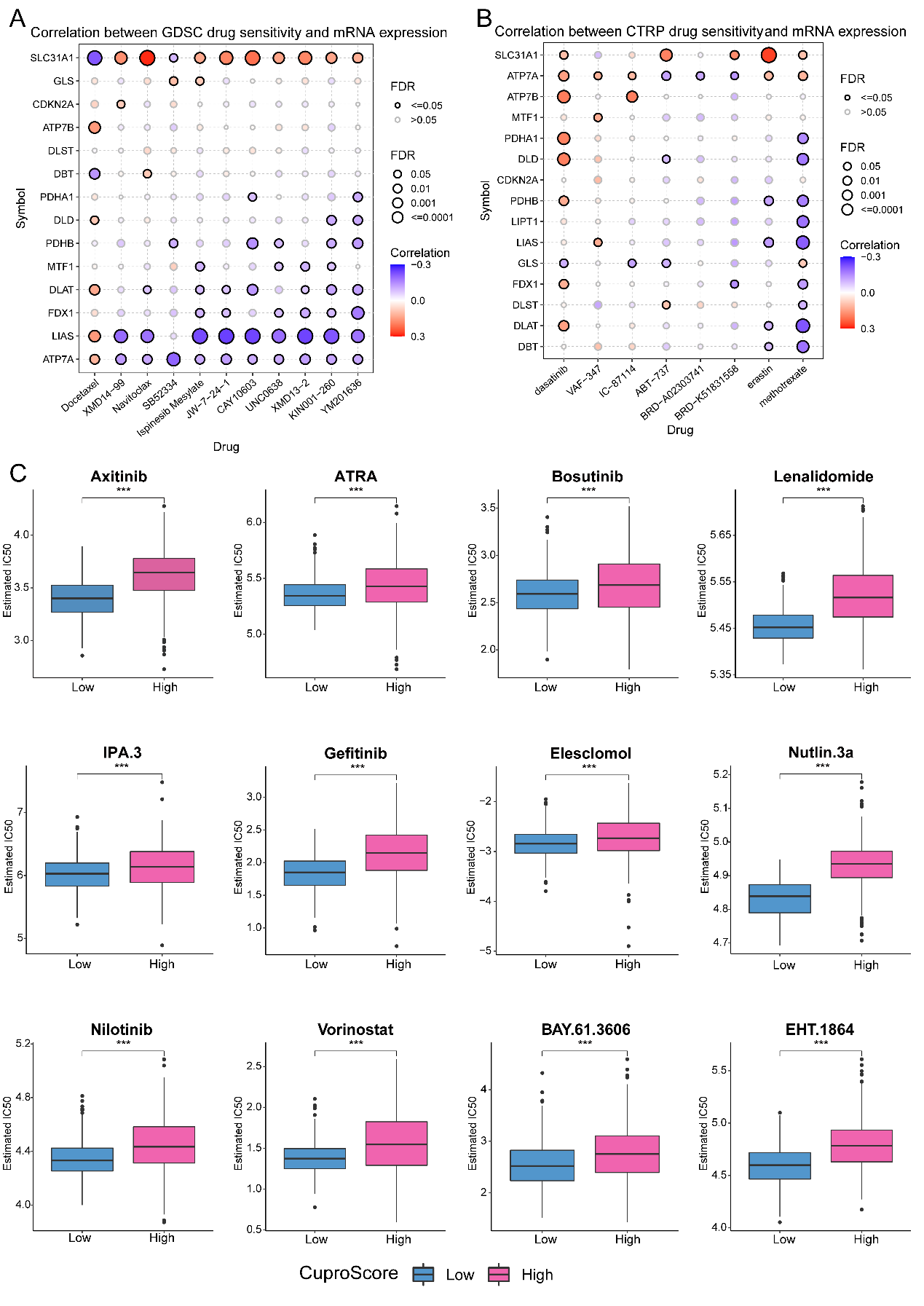


**Figure S15. CuproScore predicts drug sensitivity**

(A-B) The bubble chart shows the correlation analysis results in GDSC database (A) and CTRP database (B) of the 16 cuproptosis-related molecules on resistance to common clinical chemotherapeutics. Red indicates a positive correlation, which means that the high expression of the cuproptosis-related molecule is resistant to the drug. The opposite is valid for a negative correlation. The bubble size represents -log10(FDR). (C) The Wilcoxon test shows the difference in IC50 of common clinical chemotherapeutics between the high and low CuproScore groups.

**Supplementary Table**

Supplementary Table 1. Univariate and multivariate Cox analyses of cluster and other clinicopathologic characteristics of 11 cohorts.

Supplementary Table 2: The performance of 117 predictive models in training and testing cohorts.

Supplementary Table 3. The evaluation metrics of Cuproscore in training and testing cohorts.

Supplementary Table 4. A total of 80 published signatures were collected.
